# Characterization of circumferential antral pulmonary vein isolation areas resulting from pulsed-field catheter ablation

**DOI:** 10.1093/europace/euac111

**Published:** 2022-07-19

**Authors:** Marius Bohnen, Reinhold Weber, Jan Minners, Amir Jadidi, Martin Eichenlaub, Franz-Josef Neumann, Thomas Arentz, Heiko Lehrmann

**Affiliations:** Department of Cardiology and Angiology II (Campus Bad Krozingen), Heart Center, University Hospital Freiburg, Südring 15, 79189 Bad Krozingen, Germany; Department of Cardiology and Angiology II (Campus Bad Krozingen), Heart Center, University Hospital Freiburg, Südring 15, 79189 Bad Krozingen, Germany; Department of Cardiology and Angiology II (Campus Bad Krozingen), Heart Center, University Hospital Freiburg, Südring 15, 79189 Bad Krozingen, Germany; Department of Cardiology and Angiology II (Campus Bad Krozingen), Heart Center, University Hospital Freiburg, Südring 15, 79189 Bad Krozingen, Germany; Department of Cardiology and Angiology II (Campus Bad Krozingen), Heart Center, University Hospital Freiburg, Südring 15, 79189 Bad Krozingen, Germany; Department of Cardiology and Angiology II (Campus Bad Krozingen), Heart Center, University Hospital Freiburg, Südring 15, 79189 Bad Krozingen, Germany; Department of Cardiology and Angiology II (Campus Bad Krozingen), Heart Center, University Hospital Freiburg, Südring 15, 79189 Bad Krozingen, Germany; Department of Cardiology and Angiology II (Campus Bad Krozingen), Heart Center, University Hospital Freiburg, Südring 15, 79189 Bad Krozingen, Germany

**Keywords:** Pulmonary vein isolation, Pulsed-field ablation, Antral, Circumferential, Isolation area, Catheter ablation

## Abstract

**Aims:**

The cornerstone of pulmonary vein (PV) isolation (PVI) is a wide-area circumferential ablation (WACA) resulting in an antral PVI area. Pulsed-field ablation (PFA) is a new nonthermal ‘single-shot’ PVI technique resulting in well-characterized posterior isolation areas. However, information on circumferential PVI area is lacking. Thus, we sought to characterize the circumferential antral PVI areas after PFA-PVI.

**Methods and results:**

Atrial fibrillation (AF) patients underwent fluoroscopy-guided PVI with a pentaspline PFA catheter. Ultra-high-density voltage maps using a 20-polar circular mapping catheter were created before and immediately after PVI to identify and quantify (i) insufficient isolation areas per antral PV segment (10-segment model) and (ii) enlarged left atrial (LA) isolation areas (beyond the antral PV segments) per LA region (8-region model). The PFA-PVI with pre- (5469 ± 1822 points) and post-mapping (6809 ± 2769 points) was performed in 40 consecutive patients [age 62 ± 6 years, 25/40 (62.5%) paroxysmal AF]. Insufficient isolation areas were located most frequently in the anterior antral PV segments of the left PVs (62.5–77.5% of patients) with the largest extent (median ≥0.4 cm^2^) located in the same segments (segments 2/5/8). Enlarged LA isolation areas were located most frequently and most extensively on the posterior wall and roof region (89.5–100% of patients; median 1.1–2.7 cm^2^ per region).

**Conclusion:**

Fluoroscopy-guided PFA-PVI frequently results in insufficient isolation areas in the left anterior antral PV segments and enlarged LA isolation areas on the posterior wall/roof, which both may be extensive. To optimize the procedure, full integration of PFA catheter visualization into three-dimensional-mapping systems is needed.

## Introduction

The cornerstone of catheter ablation for atrial fibrillation (AF) is to achieve complete antral pulmonary vein isolation (PVI).^1^ Regardless of the technique used, the approach should be to create a *circumferential* isolation area specifically encompassing the antral segments of the pulmonary veins (PVs),^1^ since they frequently contain sites of AF initiation and/or maintenance. Accordingly, the effectiveness of creating a large antral isolation area [wide-area circumferential ablation (WACA)] to significantly lower AF recurrence rates has been confirmed in multiple studies.^2,3^

To achieve PVI fast and effectively, various ‘single-shot’ devices were developed, including high-intensity focused ultrasound balloon, laserballoon, and cryoballoon, of which the latter is currently the most commonly used. All of these mainly fluoroscopically guided single-shot techniques had their *circumferential* or at least *posterior* isolation area characterized.^4–6^

Pulsed-field ablation (PFA) is a new technology for cardiac catheter ablation using a nonthermal energy source, which has previously been shown to be both safe and effective.^7,8^ Efforts to define the PVI area resulting from single-shot PFA have been made, but are limited to the *posterior* PV antrum and the adjacent *posterior* wall (PW).^9,10^

The purpose of this study was to characterize the *circumferential* PVI area created by a single-shot pentaspline PFA catheter (Farawave™; Farapulse Inc., Menlo Park, CA, USA). Thus, we investigated ultra-high-density maps, acquired pre- and post-PFA-PVI, regarding frequency (qualitative analysis) and extent (quantitative analysis) of insufficient PV antral and enlarged left atrial (LA) isolation areas.

**What’s new?**

- Following fluoroscopy-guided pulmonary vein (PV) isolation (PVI) using pulsed-field ablation (PFA), ultra-high-density voltage maps post-PFA were created to characterize the resulting *circumferential* isolation area [insufficient PV antral and enlarged left atrial (LA) isolation area].
- Insufficient antral isolation occurred most frequently in anterior antral PV segments.
- The largest extent of insufficient antral isolation areas was found in the same location, but also in the anterior lower segment of the right inferior PV.
- Enlarged LA isolation areas were located most frequently and most extensively on the posterior wall and roof region of both LA sides.
- This enlarged isolation at the roof and the posterior wall on both LA sides even resulted in a connection of both low-voltage areas in 18 and 8%, respectively.
- When using PFA to achieve a circumferential antral PVI, efforts should be made to enhance anterior antral PV segment and prevent posterior wall and roof ablation.

## Methods

This was an observational, single-centre, investigator-initiated study. The work flow of the mapping and ablation procedures, as described in the following subsections, were standard of care during the selected period (September to November 2021). Analysis of the ultra-high-density maps was performed retrospectively. The study population consisted of 40 consecutive patients with paroxysmal or persistent AF who underwent their first catheter ablation procedure for antral PVI using PFA. The study protocol was reviewed and approved by our institutional review board (registration number 21-1728). All patients gave written informed consent.

Patients returned for follow-up visits in our outpatient clinic at 3 and 6 months and were assessed for arrhythmia recurrence via history, 12-lead electrocardiogram (ECG), 24 h Holter ECG, and ECGs/rhythm strips recorded at another hospital. Any antiarrhythmic drug therapy was stopped 3-month postablation. Arrhythmia recurrence was defined as documented AF, atrial flutter, or atrial tachycardia lasting longer than 30 s.

### Pulsed-field catheter ablation

The PFA system^7,8,11^ as well as the standard catheter ablation procedure including catheter placement and LA mapping have been described in detail.^2^ In brief, after acquiring the pre-PFA map, the sheath and mapping catheter were exchanged for a 13-F steerable sheath (Faradrive™; Farapulse Inc.) and a 31 or 35 mm size 12-F over-the-wire pentaspline PFA catheter (Farawave™; Farapulse Inc.). To deploy the pentaspline PFA catheter in the desired shape and to advance it into the desired antral PV area, fluoroscopy in conjunction with the three-dimensional (3D) anatomic pre-PFA map including the visualized PFA catheter electrodes was used. Baseline electrical potentials were recorded on the PFA catheter from all PVs. The PVI was performed with four paired applications (generator output 2.0 kV) per vein, resulting in two applications each in the flower and basket configuration.^7,12^ Between a pair of applications, the catheter was rotated once by 30° to assure a dense circumferential isolation area. In patients with a left common os, the superior and inferior PV branches were treated separately. Subsequently, PVI was assessed by electrograms recorded on the PFA catheter. In case of persistent PV conduction or persistent antral electrograms, additional lesions were permitted per operator discretion.

At the end of the procedure, PVI was confirmed with the 20-polar circular mapping catheter (Lasso®; Biosense Webster, USA, spacing 2-6-2, electrode size 1 mm) by demonstration of entrance and exit block. Additionally, dormant conduction with adenosine was tested. Importantly, if PVI was confirmed, no further lesions were applied regardless of any insufficient isolation revealed by the post-PFA voltage map.

### Ultra-high-density mapping

Before and after PFA-PVI, an ultra-high-density fast electroanatomic map of the LA was acquired using a 20-polar circular mapping catheter and the CARTO® 3D electroanatomic mapping system (CARTO® 3 System; Biosense Webster). In order to achieve high accuracy for electrogram acquisition, mapping was standardized utilizing the CARTO® CONFIDENSE™ module (Supplementary material online, *Table S1*). To assure precise anatomical and electrical delineation of the LA ridge, the LA appendage was explicitly not included in the maps.

### Analysis of ultra-high-density maps

The following definitions were used: First, the PV ostium was defined as the point of maximal inflection between the PV wall and the LA wall.^6,13^ Second, the PV antrum was defined as the circumferential area beginning at the PV ostium and reaching 5 mm into the LA.^14^ Third, modified from the EFFICAS I study,^15^ a 10-segment model for the left and right PV antral area was created (Supplementary material online, *Figure S1*). Forth, an eight-region model for the LA was created (Supplementary material online, *Figures S2* and *S3*). Fifth, for the purpose of comparison with previous studies,^5,6^ a peak-to-peak bipolar electrogram amplitude <0.5 mV during sinus rhythm was defined as isolation threshold. Thus, an insufficient antral PVI area was defined by a bipolar electrogram amplitude ≥0.5 mV within the antral PV segments and an enlarged LA isolation area was defined by a bipolar electrogram amplitude <0.5 mV within the LA beyond the antral PV segments.

An anatomical map without voltage data was used to outline the PV ostia, the antral PV segments (10-segment model) and the LA regions (8-region model). Thereafter, voltage data were visualized and the relevant electrograms were reviewed to assure exclusion of far-field signals, for example, of the LA appendage.

After completion, the insufficient isolation area per antral PV segment and the enlarged isolation area per LA region were measured. For each PV segment and LA region, the total area was measured as well. To further facilitate comparison with previously published data, the circumferential PVI area, total PW area, PW-PVI area, and nonablated PW area were calculated (Supplementary material online, *Table S2*).

A minimum surface area of 0.1 cm^2^ was deemed relevant and thus included in the analysis. For the assessment of enlarged LA isolation areas, both maps (pre- and post-PFA) had to be acquired in sinus rhythm or atrial pacing from the distal coronary sinus (CS) to allow reliable identification of pre-existing low-voltage areas (LVAs). All measurements were made with the CARTO® 3 software, using the ‘design line’ or ‘area measurement’ tool.

### Statistical analysis

Categorical variables are expressed as number (percentage). Continuous variables are expressed as mean ± standard deviation or median (interquartile range) as appropriate.

Within group differences were assessed using a McNemar’s test, a Wilcoxon signed rank test, or an unpaired *T* test. Between group differences were assessed using a χ^2^ test, a Mann–Whitney *U* test, or a two-sample *T* test as appropriate. All tests were two-sided, and a *P*-value of <0.05 was considered statistically significant. The SPSS Statistics 25 (IBM Corporation, Armonk, NY, USA) was used for statistical analysis.

## Results

In the 40 patients (*Table 1*) included, 38 (95%) pre-PFA maps were created in sinus rhythm/atrial pacing (distal CS), 2 (5%) in AF (total premaps 5469 ± 1822 points; *Table 1*). Of the 38 pre-PFA maps in sinus rhythm/atrial pacing, 3 (8%) patients had pre-existing LVAs on the central roof and/or anterior wall, which, however, did not interfere with the delineation of post-PFA isolation areas.

**Table 1:** Baseline and procedure characteristics (*n* = 40)

Characteristics	Value
*Baseline*	
Age (years)	62 ± 9
Male gender	28 (70)
BMI (kg/m^2^)	28 (25–29)
Hypertension	24 (60)
Diabetes	1 (2.5)
Stroke or TIA	3 (7.5)
LA diameter (mm)	41 ± 4
LVEF (%)	58 ± 4
Atrial fibrillation	
ȃParoxysmal	25 (62.5)
ȃPersistent	15 (37.5)
*Procedure*	
Procedure time (min)	110 (100–124)
Fluoro time (min)	16 (14–24)
Fluoro dose (cGcm^2^)	125 (68–196)
Premap time (min)	12 (11–16)
PFA catheter dwell time (min)	33 (28–41)
Postmap time (min)	13 (10–17)
Points premap	5469 ± 1822
Points postmap	6809 ± 2769
35 mm PFA catheter	9 (22.5)
*PV size (mm)*	
ȃLSPV	23 (20–25)
ȃLIPV	22 (20–25)
ȃRSPV	24 (21–26)
ȃRIPV	23 (20–25)
Common os	9 (22.5)

Data are displayed as *n* (%), mean ± SD, or median (IQR).

BMI, body mass index; LA, left atrium; LIPV, left inferior pulmonary vein; LSPV, left superior pulmonary vein; LVEF, left ventricular ejection fraction; PFA, pulsed-field ablation; PV, pulmonary vein; RIPV, right inferior pulmonary vein; RSPV, right superior pulmonary vein; TIA, transient ischaemic attack.

In 31/40 (77.5%) patients, the 31 mm size pentaspline PFA catheter was selected due to a maximum PV diameter of 25 (23–26) mm. Accordingly, in 9/40 (22.5%) patients, the 35 mm size PFA catheter was chosen due to a maximum PV diameter of 30 (26–32) mm (*P* = 0.003). The average number of PFA applications was 8.7 for the left superior pulmonary vein (LSPV), 8.1 for the left inferior pulmonary vein (LIPV), 8.5 for the right superior pulmonary vein, and 8.4 for the right inferior pulmonary vein (RIPV). Acute reconnection of a PV occurred only in one patient (LSPV). This PV received a second set of eight PFA applications and was thereafter isolated.

The post-PFA maps (6809 ± 2769 points) were acquired in 13 (10–17) min, all of them in sinus rhythm/atrial pacing (distal CS).

### Insufficient isolation areas: frequency (qualitative analysis)

All 160 PVs were isolated at the end of the procedure, demonstrating entrance block, exit block, and absence of dormant conduction using adenosine. Of these, 48 (30%) PVs showed a sufficient isolation in all 5 antral segments. Conversely, the remaining 112 (70%) PVs demonstrated insufficient isolation in at least one antral segment.

**Figure 1:**
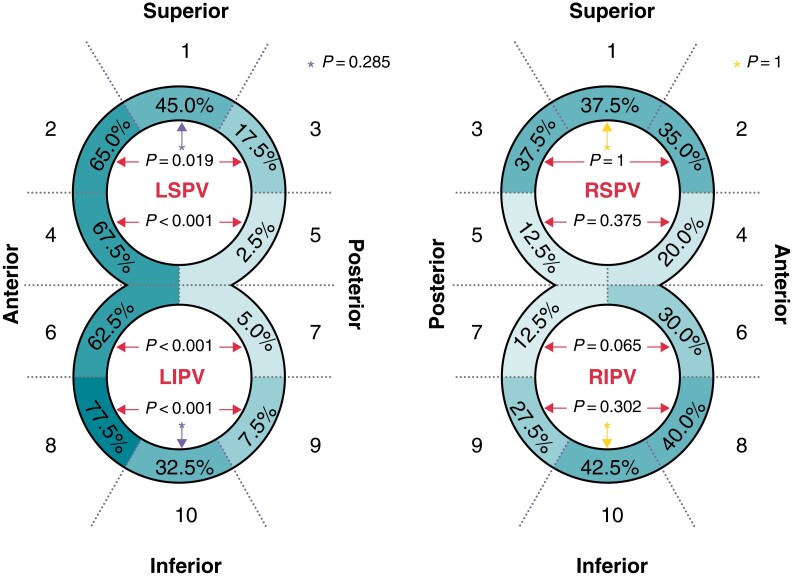
Frequency of insufficient isolation area per antral pulmonary vein segments (*n* = 40). Data are displayed as percentage. LIPV, left inferior pulmonary vein; LSPV, left superior pulmonary vein; PV, pulmonary veins; RIPV, right inferior pulmonary vein; RSPV, right superior pulmonary vein.

**Figure 2:**
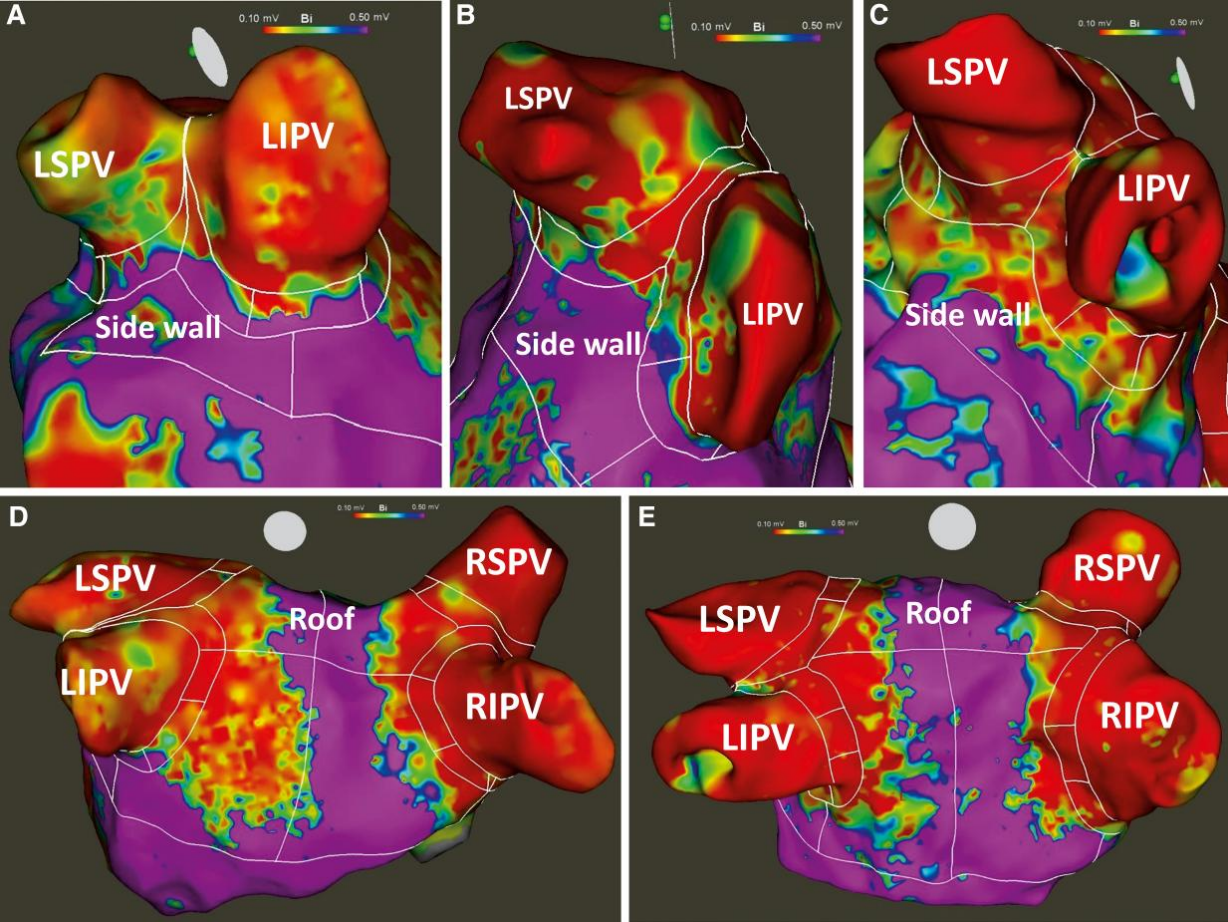
Examples of resulting isolation areas. (*A*, *B*) Insufficient isolation of the left anterior antral pulmonary vein segments. (*C*) Sufficient isolation of the left anterior antral pulmonary vein segments. (*D*) Enlarged isolation of the posterior wall. (*E*) Sufficient circumferential antral pulmonary vein isolation areas with acceptable degree of enlarged ablation. LIPV, left inferior pulmonary vein; LSPV, left superior pulmonary vein; PV, pulmonary veins; RIPV, right inferior pulmonary vein; RSPV, right superior pulmonary vein.

One-third of the overall antral PV segments had an insufficient isolation area of at least 0.1 cm^2^ [270/800 (33.75%) antral PV segments]. Furthermore, on the left side, the presence of insufficient isolation areas within the antral PV segments did depend strongly upon their location (*Table 2*, *Figure 1*). The highest rates were seen on the anterior antral PV segments (S) of the LSPV and LIPV (S 2/4/6/8; examples *Figure 2A* and *B*). On the other hand, the lowest rates (2.5–7.5%) were seen on the posterior antral PV segments of the LIPV and lower LSPV (S 5/7/9). Indeed, the anterior antral PV segments showed significantly higher rates of insufficient isolation areas than the posterior antral PV segments (*P* = 0.019, *P* < 0.001, *P* < 0.001, *P* < 0.001; *Table 2*).

**Table 2:** Qualitative analysis: frequency of insufficient and enlarged isolation areas

Frequency of *insufficient* isolation areas per antral PV segments (*n* = 40)							
PV segment		*Left PVs*			*Right PVs*		
		*n* (%)		*P*-value	*n* (%)		*P*-value
1	Sup.	18 (45.0)	*	0.285	15 (37.5)	*	1
2	Ant.	26 (65.0)	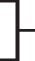	**0.019**	14 (35.0)	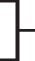	1
3	Post.	7 (17.5)			15 (37.5)		
4	Ant.	27 (67.5)	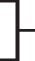	**<0.001**	8 (20.0)	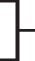	0.375
5	Post.	1 (2.5)			5 (12.5)		
6	Ant.	25 (62.5)	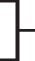	**<0.001**	12 (30.0)	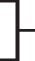	0.065
7	Post.	2 (5.0)			5 (12.5)		
8	Ant.	31 (77.5)	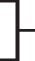	**<0.001**	16 (40.0)	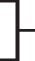	0.302
9	Post.	3 (7.5)			11 (27.5)		
10	Inf.	13 (32.5)	*	0.285	17 (42.5)	*	1
**Frequency of *enlarged* isolation areas per LA region (*n* = 38)**							
**LA region**		**Left LA**			**Right LA**		
		***n* (%)**		***P*-value**	***n* (%)**		***P*-value**
Roof		37 (97.4)	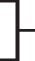	**<0.001**	34 (89.5)	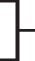	0.289
Side wall		19 (50.0)			29 (76.3)		
Posterior wall		38 (100)	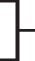	**<0.001**	35 (92.1)	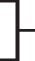	0.004
Anterior wall		20 (52.6)			26 (68.4)		

Data are displayed as *n* (%). Bold *P*-values highlight statistical significance. *Indicates comparison between PV segment 1 and 10.

LA, left atrium; PV, pulmonary vein; Sup., superior; Ant., anterior; Post., posterior; Inf., inferior.

### Insufficient isolation areas: extent (quantitative analysis)

**Figure 3:**
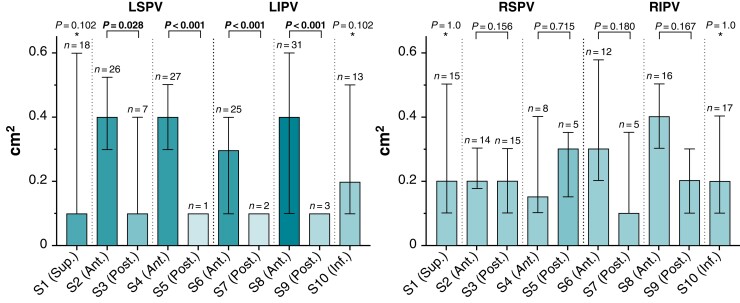
Extent of insufficient isolation area per antral *left* and *right* pulmonary vein segments (*n* = 40). Data are displayed as median (interquartile range). Bold *P* values highlight statistical significance. *Indicates comparison between PV segment 1 and 10. LIPV, left inferior pulmonary vein; LSPV, left superior pulmonary vein; PV, pulmonary veins; RIPV, right inferior pulmonary vein; RSPV, right superior pulmonary vein; S, segment.

Antral PV segments having a median of at least 0.4 cm^2^ of insufficient isolation area were almost exclusively located at the anterior aspect of the left PVs (S 2/4/8; *Table 3*, *Figure 3A* and *B*). The only exception is the anterior lower antral PV segment of the right inferior PV (S 8).

**Table 3:** Quantitative analysis: extent of insufficient, enlarged, and calculated isolation areas

Extent of *insufficient* isolation areas per antral PV segments (*n* = 40)									
Antral PV segment		Left PVs				Right PVs			
		Total area (cm^2^)	Insufficient isolation area (cm^2^)		*P*-value	Total area (cm^2^)	Insufficient isolation area (cm^2^)		*P*-value
1	Sup.	0.6 (0.5–0.7)	0.1 (0.1–0.6)	*	0.102	0.6 (0.5–0.7)	0.2 (0.1–0.5)	*	1
2	Ant.	0.6 (0.5–0.5)	0.4 (0.3–0.5)	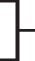	**0.028**	0.6 (0.5–0.7)	0.2 (0.2–0.3)	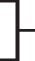	0.156
3	Post.	0.5 (0.5–0.6)	0.1 (0.1–0.4)			0.6 (0.5–0.6)	0.2 (0.1–0.3)		
4	Ant.	0.8 (0.6–1.0)	0.4 (0.3–0.5)	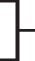	**<0.001**	0.9 (0.7–1.0)	0.2 (0.1–0.4)	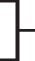	0.715
5	Post.	0.7 (0.6–0.9)	0.1 (0.1–0.1)			0.9 (0.7–1.0)	0.3 (0.2–0.4)		
6	Ant.	0.7 (0.6–0.9)	0.3 (0.1–0.4)	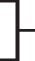	**<0.001**	0.9 (0.7–0.9)	0.3 (0.2–0.6)	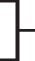	0.180
7	Post.	0.7 (0.6–0.9)	0.1 (0.1–0.1)			0.8 (0.7–1.0)	0.1 (0.1–0.4)		
8	Ant.	0.6 (0.5–0.7)	0.4 (0.1–0.6)	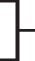	**<0.001**	0.6 (0.5–0.7)	0.4 (0.3–0.5)	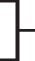	0.167
9	Post.	0.5 (0.5–0.6)	0.1 (0.1–0.1)			0.6 (0.5–0.7)	0.2 (0.1–0.3)		
10	Inf.	0.6 (0.5–0.7)	0.2 (0.1–0.5)	*	0.102	0.7 (0.6–0.7)	0.2 (0.1–0.4)	*	1
**Extent of *enlarged* isolation area per LA region (*n* = 38)**									
***LA region***		***Left LA***				***Right LA***			
		**Total area (cm^2^)**	**Enlarged isolation area (cm^2^)**		***P*-value**	**Total area (cm^2^)**	**Enlarged isolation area (cm^2^)**		***P*-value**
Roof		5.2 (4.0–6.4)	2.7 (1.3–3.5)	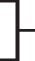	**<0.001**	4.5 (3.8–6.2)	1.4 (0.6–2.0)	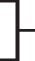	0.467
Side wall		3.6 (3.0–4.5)	0.1 (0.0–0.7)			3.8 (3.5–4.9)	1.0 (0.2–2.0)		
Posterior wall		7.8 (6.8–9.0)	2.1 (0.9–3.5)	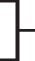	**<0.001**	7.4 (6.2–8.4)	1.1 (0.6–2.0)	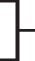	**0.002**
Anterior wall		5.7 (4.5–6.9)	0.3 (0.0–0.9)			5.9 (4.2–6.6)	0.6 (0.0–1.1)		
**Extent of *calculated* isolation areas (*n* = 38)**									
				**Total**		**Left LA**	**Right LA**	***P*-value**	
Circumferential PV isolation area (cm^2^)				22.2 ± 5.6		11.2 ± 3.3	11.0 ± 3.7	0.676	
Total posterior wall area (cm^2^)				31.2 ± 6.5		15.9 ± 3.5	15.3 ± 3.2	**0.015**	
PW-PV isolation area (cm^2^)				13.2 ± 4.0		7.7 ± 2.9	5.5 ± 2.0	**<0.001**	
Nonablated PW area (cm^2^)				18.1 ± 5.8		8.2 ± 3.2	9.8 ± 3.2	**0.001**	

Data are displayed as median (interquartile range) or mean ± standard deviation. Bold *P*-values highlight statistical significance. *Indicates comparison between PV segment 1 and 10.

Ant., anterior; Inf., inferior; LA, left atrium; Post., posterior; PV, pulmonary vein; PW, posterior wall; Sup., superior.

For the left side, anterior antral PV segments showed a significantly larger extent of insufficient isolation area than posterior antral PV segments (*P* = 0.028, *P* < 0.001, *P* < 0.001, *P* < 0.001; *Table 3*, *Figure 3A*).

### Enlarged isolation areas: frequency (qualitative analysis)

The PW and roof region of both LA sides were the most frequent locations of enlarged LA isolation areas (*Table 2*, example *Figure 2D*). For the left LA, all [38 (100%)] patients showed enlarged isolation areas on the PW and almost all [37/38 (97.4%)] patients showed enlarged isolation areas on the roof. When comparing the opposing walls on the left LA side, the PW (100%) and roof (97.4%) were significantly more likely to show enlarged isolation areas than the anterior wall (52.6%) and sidewall (50.0%), respectively (both *P* < 0.001). For the right LA side, again, the PW (92.1%) was significantly more likely to show enlarged isolation areas than the anterior wall (68.4%; *P* = 0.004).

### Enlarged isolation areas: extent (quantitative analysis)

**Figure 4:**
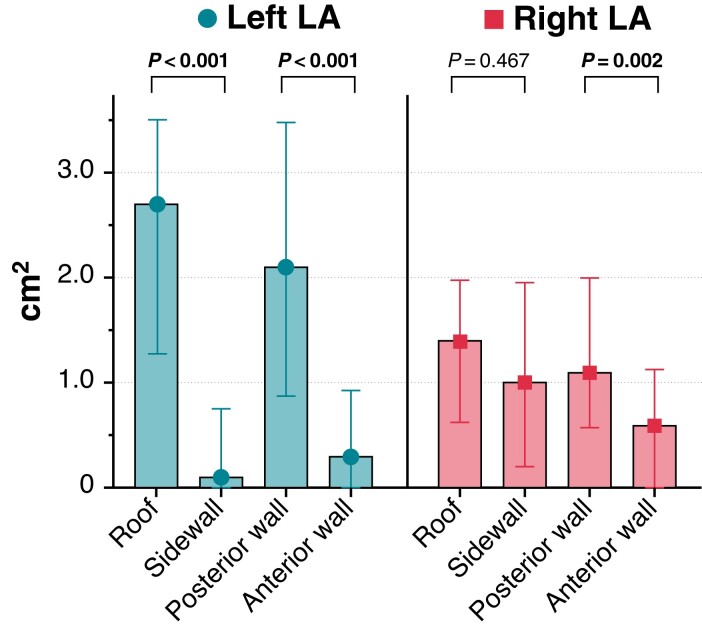
Extent of enlarged isolation area per left atrium region (*n* = 38). Data are displayed as median (interquartile range). Bold *P* values highlight statistical significance. LA, left atrium.

The left-sided roof [2.7 cm^2^ (1.3–3.5 cm^2^)] was the region with the largest extent of an enlarged LA isolation area, followed by the left-sided PW [2.1 cm^2^ (0.9–3.5 cm^2^)], the right-sided roof [1.4 cm^2^ (0.6–2.0 cm^2^)], and the right-sided PW [1.1 cm^2^ (0.6–2.0 cm^2)^; *Table 3*, *Figure 4*]. In 7/38 (18.4%) patients, the enlarged LA isolation area of both roof regions was that extensive [6.4 cm^2^ (4.7–7.3 cm^2^)], that isolation areas of both LA sides were connected [in 2/7 (29%) patients by a pre-existing LVA]. The same was seen for the PW in 3/38 (7.9%) patients [in 1/3 (33%) patients by a pre-existing LVA]. The smallest extent of an enlarged isolation area was seen on the left sidewall [0.1 cm^2^ (0.0–0.7 cm^2^)] and anterior wall [0.3 cm^2^ (0.0–0.9 cm^2^); *Table 3*, *Figure 4*].

Comparison of the opposing walls for the left-sided LA showed significantly larger LA isolation areas on the roof than on the sidewall, and similarly, significantly larger LA isolation areas on the PW than on the anterior wall (both *P* < 0.001; *Table 3*, *Figure 4*). For the right LA side, significantly larger LA isolation areas on the PW than on the anterior wall were seen (*P* = 0.002; *Table 3*).

### Extent of circumferential and posterior wall isolation areas

The circumferential PVI area for both LA sides was 22.2 ± 5.6 cm^2^ (*Table 3*). Between left- and right-sided LA, the circumferential PVI area did not differ significantly (*P* = 0.676). For the total PW area, the PW-PVI area, and the nonablated PW area, a significant difference between right and left LA sides was found (*P* = 0.015, *P* < 0.001, *P* = 0.001; *Table 3*).

### Follow-up and arrhythmia recurrence

A median of 190 days (167–202 days) of follow-up were available for all 40 patients (100%). During that time period, six patients (6/40; 15%) experienced recurrence of arrhythmia.

## Discussion

To our knowledge, this is the first study to examine the circumferential PVI area resulting from PFA. The main findings are: (i) Insufficient isolation areas were located most frequently in the anterior antral PV segments of the left PVs. (ii) The largest extent of insufficient isolation areas in the antral PV segments was located again on the anterior parts of both left PVs, but also in the anterior lower segment of the right inferior PV. (iii) On the other hand, enlarged LA isolation areas were located most frequently and most extensively on the PW and roof region of both LA sides. (iv) This enlarged isolation at the roof and the PW on both LA sides even resulted in a connection of both LVAs in 18 and 8%, respectively. (v) All PVs were acutely isolated.

### Potential reasons for insufficient and enlarged isolation areas after pulsed-field ablation pulmonary vein isolation in our study cohort

The pentaspline PFA catheter can be deployed in essentially two configurations. The basket configuration is designed for ostial ablation and is centred into the PV ostium by its shape. The flat flower configuration is capable of ablating the PV antrum; however, its position in the PV antrum is less fixed. Due to the anatomical location of the interatrial septum in relation to the LA, a trans-septally introduced catheter will align itself toward the PW, resulting in an enlarged LA ablation of this region and insufficient ablation of the anterior antral PV segments. As there is currently only very limited integration of the PFA catheter into the available mapping systems, the catheter is navigated almost exclusively by fluoroscopic guidance. A position that is slightly too posterior can therefore easily be overseen.

The observed enlarged LA isolation of the roof may be explained by guidewire positioning in superior branches of the upper PVs, which ultimately leads to alignment of the catheter system toward the roof. Simultaneously, reduced coverage of the antral PV segments on the sidewall will result.

Enlarged LA isolation found on the right sidewall is likely a result of the limited manoeuvrability of the PFA catheter due to the small distance between trans-septal puncture site and the RIPV.

Furthermore, insufficient and enlarged isolation areas can be explained by the oval shape of the PVs^16^ and the circular shape of the ablation catheter system.

### Comparison with previous data

A substantial amount of data, with respect to antral PVI area, is available for other single-shot devices for PVI, mostly the cryoballoon ablation system. Comparison of the available data is particularly limited, due to different low-voltage thresholds, varying freeze protocols, technical progress (different generations of the system), and lack of detailed analysis of the antral PV segments. The works of Miyazaki *et al*.^6^ and Nagashima *et al*.^17^ (both done with the second generation 28 mm cryoballoon) are comparable with our study cohort with respect to the low-voltage-threshold definition. In those studies, total antral PVI area was measured with 17.9 and 33.5 cm^2^, respectively. Our area of 22.2 cm^2^ is consistently in between these reported values, so that in this regard a PFA-PVI seems to be similar to a cryoballoon PVI.

Data on the isolation area after pulsed-field PVI are still scarce. Kawamura *et al*.^9,10^ published two studies with the same voltage threshold as in our study. They showed that the isolation area size did not regress between the acute (procedure end) and the chronic phase (>75 days postpulsed-field PVI).^10^ Moreover, the isolation area size was similar between the PFA-PVI cohort and a comparative thermal ablation PVI cohort (radiofrequency, cryoballoon, laserballoon).^9^ In contrast to our work, isolation area analysis was limited strictly to the PW, whereas, in our study, insufficient isolation areas were most commonly found in the anterior antral PV segments. Despite this limitation, the reported total PW ablation lesion size^9,10^ of 11 cm^2^ is compatible with our data (13 cm^2^). Interestingly, approximately 50% of the PW area was ablated, which is in line with the enlarged LA isolation area of the PW reported in our study cohort. Recently, Gunawardene *et al*.^18^ reported the isolation area in 20 patients undergoing a PFA-PVI procedure. Unfortunately, additional ablation lesions were created in 9 patients (PW isolation and/or mitral isthmus line), leaving only 11 patients with a PVI only procedure for comparison with our data. In addition, voltage maps were acquired with the Orion™ catheter and the Rhythmia™ mapping system and a different low-voltage cut-off of 0.3 mV was used. Nevertheless, the reported 44% of PW isolation area (PVI only patients) is in line with the data cited above and ours.

In the same study, in 5/80 of treated PVs, spontaneous conduction recovery occurred during the procedure.^18^ Interestingly, although no analysis of the LVAs per antral PV segment was performed, the recovery sites were located in the anterior portions of both upper PVs. Areas which our analysis identified as typical spots of insufficient isolation areas.

### Potential clinical consequences of our findings

Whether the clinical outcome is changed by an insufficient antral isolation area remains hypothetical from our work and has to be shown in larger patient cohorts. Recently published 1-year outcome data already show a high rate of PVI durability (84.8%) and clinical success rate for pulsed-field PVI (freedom from any atrial arrhythmia of 78.5%).^8^ Nevertheless, the clinical benefit of a WACA approach for PVI has been shown in several studies.^2,3^ Indisputable, recovery of PV conduction is the main reason for AF recurrence in paroxysmal AF patients.^19^ A circumferential antral PVI area will potentially prevent PV conduction recovery and should be the endpoint of every PVI procedure.^1,20^

On the other hand, enlarged LA isolation areas carry the following potential risks:

1. *Collateral damage*: Extending ablation to the LA, especially on the PW, may lead to life threatening complications in <0.05% of all PVI procedures.^1^ Thermal damage to the oesophagus, a possible precursor of atrioesophageal fistula, has been reported in 11% of patients following a conventional RF-PVI procedure.^21^ In contrast, in small clinical PFA-PVI trials, no oesophageal late gadolinium enhancement on MRI^8,22^ or thermal lesions on oesophagoscopy^8^ have been detected so far.
2. *Damage to contractile atrial tissue*: Enlarged LA thermal ablation can lead to scarring and loss of atrial contractility. However, this was not observed in a recent paper for PFA procedures.^23^
3. *Risk for atrial tachycardia*: Conventional thermal ablation energy leads to ‘unorganized’ fibrotic scarring and may increase the risk for atrial tachycardia.^18^ In early clinical PFA-PVI data, only very small areas of fractionated atrial signals have been found in the ablation margins after PVI.^18^ Whether this translates into reduced risk for atrial tachycardia post-PVI has to be studied in a larger patient cohorts. In our study, connecting lesions at the LA roof or the PW occurred in 18 and 8%, respectively. Apart from fractionated atrial signals, connecting lesions without conduction block or recovery of block may ultimately lead to macro-re-entry tachycardia and should therefore be avoided.

### Limitations

In our study, the PFA catheter was selected based on the PV size. In most cases, the small catheter (31 mm) was used. Thus, we cannot make any reliable statements about the influence of catheter size on the resulting isolation area.

The presented data are only applicable for the PFA system (Farapulse™) used in our study. Other systems, with for example a different catheter shape, electrode orientation, energy output, waveform, pulse architecture, amplitude, or duration, may behave completely different.

Since we do not routinely use voltage-mapping in other single-shot PVI procedures (e.g. cryoballoon PVI), we can only provide indirect comparison of the PV antral isolation area between different techniques (see Discussion section for details). Further comparative prospective studies are needed to analyse in detail the differences in insufficient and extensive PVI areas between the available single-shot PVI devices.

## Conclusion

During mainly fluoroscopy-guided pulsed-field PVI procedures, insufficient antral isolation occurred most frequently in anterior antral PV segments. The largest extent of insufficient antral isolation areas was found in the same location, but also in the anterior lower segment of the right inferior PV. Enlarged LA isolation areas were located most frequently and most extensively on the PW and roof region of both LA sides. This enlarged isolation at the roof and the PW on both LA sides even resulted in a connection of both LVAs in 18 and 8%, respectively.

When using PFA to achieve a circumferential antral PVI, efforts should be made to enhance anterior antral PV segment and prevent PW and roof ablation. To further optimize the procedure, full integration of PFA catheter visualization into 3D-mapping systems is needed.

## Supplementary material

Supplementary material is available at *Europace* online.

## Supplementary Material

euac111_Supplementary_Data

## Data Availability

Data are available on request to the corresponding author.
