# Supplementary material for: Characterization of circumferential antral pulmonary vein isolation areas resulting from pulsed-field catheter ablation
Source: Europace. 2022 Jul 19;25(1):65–73. doi: 10.1093/europace/euac111 (PMC10103571; doi:10.1093/europace/euac111)
Supplement: euac111_Supplementary_Data [file euac111_supplementary_data.zip › Manuscript Rv2 - EUPC-D-22-00235 Tracked Changes.docx]

**Title:** **Characterization of Circumferential Antral Pulmonary Vein Isolation Areas resulting from Pulsed-Field Catheter Ablation**

**Short title:** Characterization of Circumferential Antral PFA-PVI Area

**Authors:** Marius Bohnen, MD; Reinhold Weber, MD; Jan Minners, MD, PhD; Amir Jadidi, MD; Martin Eichenlaub, MD; Franz-Josef Neumann, MD; Thomas Arentz, MD; Heiko Lehrmann, MD

**Affiliation:** Department of Cardiology and Angiology II (Campus Bad Krozingen), Heart Center, University Hospital Freiburg, Germany

**Corresponding author:** Marius Bohnen, MD; Heart Center, University Hospital Freiburg; Department of Cardiology and Angiology II (Campus Bad Krozingen); Südring 15; 79189 Bad Krozingen; Phone: +49 7633 402 0; Email: [marius.bohnen@uniklinik-freiburg.de](mailto:marius.bohnen@uniklinik-freiburg.de)

**Word count:** 3492

**Funding:** This research did not receive any specific grant from funding agencies in the public, commercial, or not-for-profit sectors.

**Introduction**

The cornerstone of catheter ablation for atrial fibrillation (AF) is to achieve complete antral pulmonary vein isolation (PVI).^1^ Regardless of the technique used, the approach should be to create a *circumferential* isolation area specifically encompassing the antral segments of the pulmonary veins (PV)^1^ since they frequently contain sites of AF initiation and/or maintenance. Accordingly, the effectiveness of creating a large antral isolation area (wide-area *circumferential* ablation [WACA]) to significantly lower AF recurrence rates has been confirmed in multiple studies.^2,3^

To achieve PVI fast and effectively, various “single-shot” devices were developed, including high-intensity focused ultrasound balloon, laserballoon, and cryoballoon, of which the latter is currently the most commonly used. All of these mainly fluoroscopically guided “single-shot” techniques had their *circumferential* or at least *posterior* isolation area characterized .^4–6^

Pulsed field ablation (PFA) is a new technology for cardiac catheter ablation using a non-thermal energy source, which has previously been shown to be both safe and effective.^7,8^ Efforts to define the PV isolation area resulting from “single-shot” PFA have been made, but are limited to the *posterior* PV antrum and the adjacent *posterior* wall.^9,10^

The purpose of this study was to characterize the *circumferential* PV isolation area created by a “single-shot” pentaspline PFA catheter (Farawave^TM^, Farapulse Inc., Menlo Park CA, USA). Thus, we investigated ultra-high-density maps, acquired pre- and post PFA-PVI, regarding frequency (qualitative analysis) and extent (quantitative analysis) of insufficient PV antral and enlarged LA isolation areas.

**Methods**

This was an observational, single-center, investigator-initiated study. The work flow of the mapping and ablation procedures, as described in the following subsections, were standard of care during the selected period (September to November 2021). Analysis of the ultra-high-density maps was performed retrospectively. The study population consisted of forty consecutive patients with paroxysmal or persistent AF who underwent their first catheter ablation procedure for antral PVI using PFA. The study protocol was reviewed and approved by our institutional review board (registration number 21-1728). All patients gave written informed consent.

Patients returned for follow-up visits in our outpatient clinic at 3 and 6 months and were assessed for arrhythmia recurrence via history, 12 lead ECG, 24h holter ECG and ECGs/rhythm strips recorded at another hospital. Any antiarrhythmic drug therapy was stopped 3-month post ablation. Arrhythmia recurrence was defined as documented atrial fibrillation, atrial flutter, or atrial tachycardia lasting longer than 30 seconds.

Pulsed-Field Catheter Ablation

The PFA system^7,8,11^ as well as the standard catheter ablation procedure including catheter placement and left atrial mapping have been described in detail.^2^ In brief, after acquiring the pre PFA map, the sheath and mapping catheter were exchanged for a 13-F steerable sheath (Faradrive^TM^, Farapulse Inc., Menlo Park, CA, USA) and a 31 mm or 35 mm size 12-F over-the-wire pentaspline PFA catheter (Farawave^TM^, Farapulse Inc.). To deploy the pentaspline PFA catheter in the desired shape and to advance it into the desired antral PV area, fluoroscopy in conjunction with the three-dimensional anatomic pre PFA map including the visualized PFA catheter electrodes was used. Baseline electrical potentials were recorded on the PFA catheter from all PVs. PVI was performed with 4 paired applications (generator output 2.0 kV) per vein, resulting in 2 applications each in the flower and basket configuration.^7,12^ Between a pair of applications, the catheter was rotated once by 30° to assure a dense circumferential isolation area. In patients with a left common os, the superior and inferior PV branch were treated separately. Subsequently, PVI was assessed by electrograms recorded on the PFA catheter. In case of persistent PV conduction or persistent antral electrograms, additional lesions were permitted per operator discretion.

At the end of the procedure, PVI was confirmed with the 20-polar circular mapping catheter (Lasso®, Biosense Webster, USA, spacing 2-6-2, electrode size 1 mm) by demonstration of entrance and exit block. Additionally, dormant conduction with adenosine was tested. Importantly, if PVI was confirmed, no further lesions were applied regardless of any insufficient isolation revealed by the post PFA voltage map.

Ultra-High-Density Mapping

Before and after PFA-PVI, an ultra-high-density fast electroanatomic map of the left atrium (LA) was acquired using a 20-polar circular mapping catheter and the CARTO® 3D electroanatomic mapping system (CARTO® 3 System, Biosense Webster, USA). In order to achieve high accuracy for electrogram acquisition, mapping was standardized utilizing the CARTO® CONFIDENSE™ module (Suppl. Table 1). To assure precise anatomical and electrical delineation of the left atrial ridge, the left atrial appendage was explicitly not included in the maps.

Analysis of Ultra-High-Density Maps

The following definitions were used: First, the PV ostium was defined as the point of maximal inflection between the PV wall and the LA wall.^6,13^ Second, the PV antrum was defined as the circumferential area beginning at the PV ostium and reaching 5 mm into the LA.^14^ Third, modified from the EFFICAS I study,^15^ a 10-segment model for the left and right PV antral area was created (Suppl. Figure 1). Forth, an 8-region model for the LA was created (Suppl. Figure 2 and 3). Fifth, for the purpose of comparison with previous studies,^5,6^ a peak-to-peak bipolar electrogram amplitude < 0.5 mV during sinus rhythm was defined as isolation threshold. Thus, an insufficient antral PV isolation area was defined by a bipolar electrogram amplitude ≥ 0,5 mV within the antral PV segments and an enlarged LA isolation area was defined by a bipolar electrogram amplitude < 0,5 mV within the LA beyond the antral PV segments.

An anatomical map without voltage data was used to outline the PV ostia, the antral PV segments (10-segment model) and the LA regions (8-region model). Thereafter, voltage data was visualized and the relevant electrograms were reviewed to assure exclusion of far-field signals, for example of the left atrial appendage.

After completion, the insufficient isolation area per antral PV segment and the enlarged isolation area per LA region were measured. For each PV segment and LA region, the total area was measured as well. To further facilitate comparison with previously published data, the circumferential PV isolation area, total posterior wall (PW) area, PW PV isolation area, and non-ablated PW area were calculated (Suppl. Table 2).

A minimum surface area of 0.1 cm² was deemed relevant and thus included in the analysis. For the assessment of enlarged LA isolation areas, both maps (pre PFA and post PFA) had to be acquired in sinus rhythm or atrial pacing from the distal coronary sinus (CS) to allow reliable identification of preexisting low voltage areas. All measurements were made with the CARTO® 3 software, using the “design line” or “area measurement” tool.

Statistical Analysis

Categorical variables are expressed as number (percentage). Continuous variables are expressed as mean ± standard deviation or median (interquartile range) as appropriate.

Within group differences were assessed using a McNemar's test, a Wilcoxon Signed Rank test, or an unpaired T test. Between group differences were assessed using a Chi Square test, a Mann Whitney U test, or a 2 sample T test as appropriate. All tests were 2-sided, and a P-value of < 0,05 was considered statistically significant. SPSS Statistics 25 (IBM Corporation, Armonk, NY, USA) was used for statistical analysis.

**Results**

In the 40 patients (Table 1) included, thirty-eight (95%) pre PFA maps were created in sinus rhythm/atrial pacing (distal CS), two (5%) in AF (total pre maps 5469±1822 points; Table 1). Of the 38 pre PFA maps in sinus rhythm/atrial pacing, three (8%) patients had preexisting low voltage areas on the central roof and/or anterior wall, which, however, did not interfere with the delineation of post PFA isolation areas.

In 31/40 (77.5%) patients, the 31 mm size pentaspline PFA catheter was selected due to a maximum PV diameter of 25 (23-26) mm. Accordingly, in 9/40 (22.5%) patients, the 35 mm size PFA catheter was chosen due to a maximum PV diameter of 30 (26-32) mm (p=0.003). The average number of PFA applications was 8.7 for the LSPV, 8.1 for the LIPV, 8.5 for the RPSV, and 8.4 for the RIPV. Acute reconnection of a PV occurred only in one patient (LSPV). This PV received a second set of 8 PFA applications and was thereafter isolated.

The post PFA maps (6809±2769 points) were acquired in 13 (10-17) minutes, all of them in sinus rhythm/atrial pacing (distal CS).

Insufficient Isolation Areas – Frequency (Qualitative Analysis)

All 160 pulmonary veins (PVs) were isolated at the end of the procedure, demonstrating entrance block, exit block, and absence of dormant conduction using adenosine. Of these, 48 (30%) PVs showed a sufficient isolation in all 5 antral segments. Conversely, the remaining 112 (70%) PVs demonstrated insufficient isolation in at least one antral segment.

One-third of the overall antral PV segments had an insufficient isolation area of at least 0.1 cm² (270/800 [33.75%] antral PV segments). Furthermore, on the left side, the presence of insufficient isolation areas within the antral PV segments did depend strongly upon their location (Table 2A, Figure 1). The highest rates were seen on the anterior antral PV segments (S) of the LSPV and LIPV (S 2/4/6/8; examples Figure 2A+B). On the other hand, the lowest rates (2.5-7.5%) were seen on the posterior antral PV segments of the LIPV and lower LSPV (S 5/7/9). Indeed, the anterior antral PV segments showed significantly higher rates of insufficient isolation areas than the posterior antral PV segments (p=0.019, p<0.001, p<0.001, p<0.001; Table 2A).

Insufficient Isolation Areas – Extent (Quantitative Analysis)

Antral PV segments having a median of at least 0.4 cm² of insufficient isolation area were almost exclusively located at the anterior aspect of the left PVs (S 2/4/8; Table 3A; Figure 3AB). The only exception being the anterior lower antral PV segment of the right inferior PV (S 8).

For the left side, anterior antral PV segments showed a significantly larger extent of insufficient isolation area than posterior antral PV segments (p=0.028, p<0.001, p<0.001, p<0.001; Table 3A, Figure 3A).

Enlarged Isolation Areas – Frequency (Qualitative Analysis)

The posterior wall and roof region of both LA sides were the most frequent locations of enlarged LA isolation areas (Table 2A; example Figure 2D). For the left LA, all (38 [100%]) patients showed enlarged isolation areas on the posterior wall and almost all (37/38 [97.4%]) patients showed enlarged isolation areas on the roof. When comparing the opposing walls on the left LA side, the posterior wall (100%) and roof (97.4%) were significantly more likely to show enlarged isolation areas than the anterior wall (52.6%) and sidewall (50.0%), respectively (both p<0.001). For the right LA side, again, the posterior wall (92.1%) was significantly more likely to show enlarged isolation areas than the anterior wall (68.4%; p=0.004).

Enlarged Isolation Areas – Extent (Quantitative Analysis)

The left sided roof (2.7 cm² [1.3-3.5 cm²]) was the region with the largest extent of an enlarged LA isolation area, followed by the left sided posterior wall (2.1 cm² [0.9-3.5 cm²]), the right sided roof (1.4 cm² [0.6-2.0 cm²]), and the right sided posterior wall (1.1 cm² [0.6-2.0 cm²]; Table 3B; Figure 4). In 7/38 (18.4%) patients, the enlarged LA isolation area of both roof regions was that extensive (6.4 cm² [4.7-7.3 cm²]), that isolation areas of both LA sides were connected (in 2/7 (29%) patients by a preexisting low voltage area [LVA]). The same was seen for the posterior wall in 3/38 (7.9%) patients (in 1/3 [33%] patients by a preexisting LVA). The smallest extent of an enlarged isolation area was seen on the left sidewall (0.1 cm² [0.0-0.7 cm²]) and anterior wall (0.3 cm² [0.0-0.9 cm²]; Table 3B; Figure 4).

Comparison of the opposing walls for the left sided LA showed significantly larger LA isolation areas on the roof than on the sidewall, and similarly, significantly larger LA isolation areas on the posterior wall than on the anterior wall (both p<0.001; Table 3B; Figure 4). For the right LA side, significantly larger LA isolation areas on the posterior wall than on the anterior wall were seen (p=0.002; Table 3B).

Extent of Circumferential and Posterior Wall Isolation Areas

The circumferential PV isolation area for both LA sides was 22.2 ± 5.6 cm² (Table 3C). Between left and right-sided LA, the circumferential PV isolation area did not differ significantly (p=0.676). For the total posterior wall area, the PW PV isolation area, and the non-ablated PW area, a significant difference between right and left LA side was found (p=0.015, p<0.001, p=0.001; Table 3C).

Follow-Up and Arrhythmia Recurrence

A median of 190 days (167-202 days) of follow-up were available for all 40 patients (100%). During that time period, six patients (6/40; 15%) experienced recurrence of arrhythmia.

**Discussion**

To our knowledge, this is the first study to examine the circumferential PV isolation area resulting from pulsed field ablation. The main findings are: (I) Insufficient isolation areas were located most frequently in the anterior antral PV segments of the left PVs. (II) The largest extent of insufficient isolation areas in the antral PV segments was located again on the anterior parts of both left PVs, but also in the anterior lower segment of the right inferior PV. (III) On the other hand, enlarged LA isolation areas were located most frequently and most extensively on the posterior wall and roof region of both LA sides. (IV) This enlarged isolation at the roof and the posterior wall on both LA sides even resulted in a connection of both low voltage areas in 18% and 8%, respectively. (V) All PVs were acutely isolated.

Potential Reasons for Insufficient and Enlarged Isolation Areas after PFA-PVI in our Study Cohort

The pentaspline PFA catheter can be deployed in essentially two configurations. The basket configuration is designed for ostial ablation and is centered into the PV ostium by its shape. The flat flower configuration is capable of ablating the PV antrum, however, its position in the PV antrum is less fixed. Due to the anatomical location of the interatrial septum in relation to the left atrium, a transseptally introduced catheter will align itself towards the posterior wall, resulting in an enlarged LA ablation of this region and insufficient ablation of the anterior antral PV segments. As there is currently only very limited integration of the PFA catheter into the available mapping systems, the catheter is navigated almost exclusively by fluoroscopic guidance. A position that is slightly too posterior can therefore easily be overseen.

The observed enlarged LA isolation of the roof may be explained by guidewire positioning in superior branches of the upper PVs, which ultimately leads to alignment of the catheter system towards the roof. Simultaneously, reduced coverage of the antral PV segments on the sidewall will result.

Enlarged LA isolation found on the right sidewall is likely a result of the limited maneuverability of the PFA catheter due to the small distance between transseptal puncture site and the RIPV.

Furthermore, insufficient and enlarged isolation areas can be explained by the oval shape of the PVs^16^ and the circular shape of the ablation catheter system.

Comparison with previous data

A substantial amount of data, with respect to antral PV isolation area, is available for other “single shot” devices for PVI, mostly the cryoballoon ablation system. Comparison of the available data is particularly limited, due to different low-voltage thresholds, varying freeze protocols, technical progress (different generations of the system) and lack of detailed analysis of the antral PV segments. The works of Miyazaki et al.^6^ and Nagashima et al.^17^ (both done with the 2^nd^ generation 28 mm cryoballoon) are comparable to our study cohort with respect to the low-voltage-threshold definition. In those studies, total antral PV isolation area was measured with 17.9 cm^2^ and 33.5 cm^2^, respectively. Our area of 22.2 cm^2^ is consistently in between these reported values, so that in this regard a PFA-PVI seems to be similar to a cryoballoon PVI.

Data on the isolation area after pulsed-field PVI are still scarce. Kawamura et al. published two studies with the same voltage threshold as in our study.^9,10^ They showed that the isolation area size did not regress between the acute (procedure end) and the chronic phase (>75 days post pulsed-field PVI).^10^ Moreover, the isolation area size was similar between the PFA-PVI cohort and a comparative thermal ablation PVI cohort (radiofrequency, cryoballoon, laserballoon).^9^ In contrast to our work, isolation area analysis was limited strictly to the posterior wall, whereas, in our study, insufficient isolation areas were most commonly found in the anterior antral PV segments. Despite this limitation, the reported total posterior wall ablation lesion size^9,10^ of 11 cm^2^ is compatible with our data (13 cm^2^). Interestingly, approximately 50% of the posterior wall area was ablated, which is in line with the enlarged LA isolation area of the posterior wall reported in our study cohort. Recently, Gunawardene et al.^18^ reported the isolation area in 20 patients undergoing a PFA-PVI procedure. Unfortunately, additional ablation lesions were created in 9 patients (posterior wall isolation and/or mitral isthmus line), leaving only 11 patients with a PVI only procedure for comparison with our data. In addition, voltage maps were acquired with the Orion™ catheter and the Rhythmia™ mapping system and a different low voltage cut-off of 0.3 mV was used. Nevertheless, the reported 44% of posterior wall isolation area (PVI only patients) is in line with the data cited above and ours.

In the same study, in 5/80 of treated PVs, spontaneous conduction recovery occurred during the procedure.^18^ Interestingly, although no analysis of the low-voltage areas per antral PV segment was performed, the recovery sites were located in the anterior portions of both upper PVs. Areas which our analysis identified as typical spots of insufficient isolation areas.

Potential clinical consequences of our findings

Whether the clinical outcome is changed by an **insufficient** antral isolation area remains hypothetical from our work and has to be shown in larger patient cohorts. Recently published 1-year outcome data already show a high rate of PVI durability (84.8%) and clinical success rate for pulsed field PVI (freedom from any atrial arrhythmia of 78.5%).^19^ Nevertheless, the clinical benefit of a wide antral circumferential ablation (WACA) approach for PVI has been shown in several studies.^2,3^ Indisputable, recovery of PV conduction is the main reason for AF recurrence in paroxysmal AF patients.^20^ A circumferential antral PV isolation area will potentially prevent PV conduction recovery and should be the endpoint of every PVI procedure.^1,21^

On the other hand, **enlarged** LA isolation areas carry the following potential risks:

1. *Collateral damage:* Extending ablation to the LA, especially on the posterior wall, may lead to life threatening complications in <0.05% of all PVI procedures.^1^ Thermal damage to the esophagus, a possible precursor of atrioesophageal fistula, has been reported in 11% of patients following a conventional RF-PVI procedure.^22^ In contrast, in small clinical PFA-PVI trials, no esophageal late gadolinium enhancement on MRI^8,23^ or thermal lesions on esophagoscopy^8^ have been detected so far.
2. *Damage to contractile atrial tissue:* Enlarged LA thermal ablation can lead to scarring and loss of atrial contractility. However, this was not observed in a recent paper for PFA procedures.^24^
3. *Risk for atrial tachycardia:* Conventional thermal ablation energy leads to “unorganized” fibrotic scarring and may increase the risk for atrial tachycardia.^18^ In early clinical PFA-PVI data, only very small areas of fractionated atrial signals have been found in the ablation margins after PVI.^18^ Whether this translates into reduced risk for atrial tachycardia post PVI has to be studied in a larger patient cohorts. In our study, connecting lesions at the left atrial roof or the posterior wall occurred in 18% and 8%, respectively. Apart from fractionated atrial signals, connecting lesions without conduction block or recovery of block may ultimately lead to macro-reentry tachycardia and should therefore be avoided.

Limitations

In our study, the pulsed-field ablation catheter was selected based on the PV size. In most cases the small catheter (31 mm) was used. Thus, we cannot make any reliable statements about the influence of catheter size on the resulting isolation area.

The presented data are only applicable for the pulsed-field ablation system (Farapulse™) used in our study. Other systems, with for example a different catheter shape, electrode orientation, energy output, waveform, pulse architecture, amplitude or duration, may behave completely different.

Since we do not routinely use voltage-mapping in other “single-shot” PVI procedures (e.g. cryoballoon PVI), we can only provide indirect comparison of the PV antral isolation area between different techniques (see discussion section for details). Further comparative prospective studies are needed to analyze in detail the differences in insufficient and extensive PV isolation areas between the available “single shot” PVI devices.

Conclusion

During mainly fluoroscopy-guided pulsed-field PVI procedures, insufficient antral isolation occurred most frequently in anterior antral PV segments. The largest extent of insufficient antral isolation areas was found in the same location, but also in the anterior lower segment of the right inferior PV. Enlarged LA isolation areas were located most frequently and most extensively on the posterior wall and roof region of both LA sides. This enlarged isolation at the roof and the posterior wall on both LA sides even resulted in a connection of both low voltage areas in 18% and 8%, respectively.

When using PFA to achieve a circumferential antral PVI, efforts should be made to enhance anterior antral PV segment and prevent posterior wall and roof ablation. To further optimize the procedure, full integration of PFA catheter visualization into 3D-mapping systems is needed.

**Conflict of interest statement**

Marius Bohnen reports educational grant support from Boston Scientific (Fellowship “Herzrhythmus”).

Franz-Josef Neumann reports lecture fees paid to his institution from Amgen, Bayer Healthcare, Boehringer Ingelheim, Boston Scientific, Daiichi Sankyo, Edwards Lifesciences, Ferrer, Pfizer, Novartis; consultancy fees paid to his institution from Boehringer Ingelheim, Novartis and grant support from Bayer Healthcare, Boston Scientific, Biotronik, Edwards Lifesciences, GlaxoSmithKline, Medtronic, Pfizer, Abbot Vascular.

All remaining authors have declared no conflicts of interest.

**References**

1. Hindricks G, Potpara T, Dagres N, Arbelo E, Bax JJ, Blomström-Lundqvist C, *et al.* 2020 ESC Guidelines for the diagnosis and management of atrial fibrillation developed in collaboration with the European Association for Cardio-Thoracic Surgery (EACTS). *Eur Heart J* 2021;**42**:373–498.

2. Arentz T, Weber R, Bürkle G, Herrera C, Blum T, Stockinger J, *et al.* Small or large isolation areas around the pulmonary veins for the treatment of atrial fibrillation? Results from a prospective randomized study. *Circulation* 2007;**115**:3057–63.

3. Kiuchi K, Kircher S, Watanabe N, Gaspar T, Rolf S, Arya A, *et al.* Quantitative analysis of isolation area and rhythm outcome in patients with paroxysmal atrial fibrillation after circumferential pulmonary vein antrum isolation using the pace-and-ablate technique. *Circ Arrhythmia Electrophysiol* 2012;**5**:667–75.

4. Chierchia GB, Asmundis C De, Sorgente A, Paparella G, Sarkozy A, Müller-Burri SA, *et al.* Anatomical extent of pulmonary vein isolation after cryoballoon ablation for atrial fibrillation: Comparison between the 23 and 28 mm balloons. *J Cardiovasc Med* 2011;**12**:162–6.

5. Kenigsberg DN, Martin N, Lim HW, Kowalski M, Ellenbogen KA. Quantification of the cryoablation zone demarcated by pre- and postprocedural electroanatomic mapping in patients with atrial fibrillation using the 28-mm second-generation cryoballoon. *Heart Rhythm*; 2015;**12**:283–90.

6. Miyazaki S, Taniguchi H, Hachiya H, Nakamura H, Takagi T, Iwasawa J, *et al.* Quantitative Analysis of the Isolation Area During the Chronic Phase After a 28-mm Second-Generation Cryoballoon Ablation Demarcated by High-Resolution Electroanatomic Mapping. *Circ Arrhythmia Electrophysiol* 2016;**9**:1–9.

7. Reddy VY, Neuzil P, Koruth JS, Petru J, Funosako M, Cochet H, *et al.* Pulsed Field Ablation for Pulmonary Vein Isolation in Atrial Fibrillation. *J Am Coll Cardiol* 2019;**74**.

8. Reddy VY, Dukkipati SR, Neuzil P, Anic A, Petru J, Funasako M, *et al.* Pulsed Field Ablation of Paroxysmal Atrial Fibrillation: 1-Year Outcomes of IMPULSE, PEFCAT, and PEFCAT II. *JACC Clin Electrophysiol*.; 2021;**7**:614–27.

9. Kawamura I, Neuzil P, Shivamurthy P, Kuroki K, Lam J, Musikantow D, *et al.* How does the level of pulmonary venous isolation compare between pulsed field ablation and thermal energy ablation (radiofrequency, cryo, or laser)? *Europace*;**23**:1757–66.

10. Kawamura I, Neuzil P, Shivamurthy P, Petru J, Funasako M, Minami K, *et al.* Does pulsed field ablation regress over time? A quantitative temporal analysis of pulmonary vein isolation. *Heart Rhythm*; 2021;**18**:878–84.

11. Reddy VY, Anic A, Koruth J, Petru J, Funasako M, Minami K, *et al.* Pulsed Field Ablation in Patients With Persistent Atrial Fibrillation. *J Am Coll Cardiol* 2020;**76**:1068–80.

12. Reddy VY, Koruth J, Jais P, Petru J, Timko F, Skalsky I, *et al.* Ablation of Atrial Fibrillation With Pulsed Electric Fields: An Ultra-Rapid, Tissue-Selective Modality for Cardiac Ablation. *JACC Clin Electrophysiol* 2018;**4**:987–95.

13. Dukkipati SR, Cuoco F, Kutinsky I, Aryana A, Bahnson TD, Lakkireddy D, *et al.* Pulmonary Vein Isolation Using the Visually Guided Laser Balloon: A Prospective, Multicenter, and Randomized Comparison to Standard Radiofrequency Ablation. *J Am Coll Cardiol*; 2015;**66**:1350–60.

14. Rodríguez-Mañero M, Valderrábano M, Baluja A, Kreidieh O, Martínez-Sande JL, García-Seara J, *et al.* Validating Left Atrial Low Voltage Areas During Atrial Fibrillation and Atrial Flutter Using Multielectrode Automated Electroanatomic Mapping. *JACC Clin Electrophysiol* 2018;**4**:1541–52.

15. Neuzil P, Reddy VY, Kautzner J, Petru J, Wichterle D, Shah D, *et al.* Electrical reconnection after pulmonary vein isolation is contingent on contact force during initial treatment: Results from the EFFICAS I study. *Circ Arrhythmia Electrophysiol*; 2013;**6**:327–33.

16. Ahmed J, Sohal S, Malchano ZJ, Holmvang G, Ruskin JN, Reddy VY. Three-dimensional analysis of pulmonary venous ostial and antral anatomy: Implications for balloon catheter-based pulmonary vein isolation. *J Cardiovasc Electrophysiol* 2006;**17**:251–5.

17. Nagashima K, Okumura Y, Watanabe I, Nakahara S, Hori Y, Iso K, *et al.* Hot Balloon Versus Cryoballoon Ablation for Atrial Fibrillation: Lesion Characteristics and Middle-Term Outcomes. *Circ Arrhythm Electrophysiol*; 2018;**11**:e005861–e005861.

18. Gunawardene MA, Schaeffer BN, Jularic M, Eickholt C, Maurer T, Akbulak RÖ, *et al.* Pulsed field ablation combined with ultra‐high‐density mapping in patients undergoing catheter ablation for atrial fibrillation: practical and electrophysiological considerations. *J Cardiovasc Electrophysiol* 2022;

19. Reddy VY, Dukkipati SR, Neuzil P, Anic A, Petru J, Funasako M, *et al.* Pulsed Field Ablation of Paroxysmal Atrial Fibrillation: 1-Year Outcomes of IMPULSE, PEFCAT, and PEFCAT II. *JACC Clin Electrophysiol* 2021;**7**:614–27.

20. Ouyang F, Antz M, Ernst S, Hachiya H, Mavrakis H, Deger FT, *et al.* Recovered pulmonary vein conductiona as a dominant factor for recurrent atrial tachyarrhythmias after complete circular isolation of the pulmonary veins: Lessons from double lasso technique. *Circulation* 2005;**111**:127–35.

21. Calkins H, Hindricks G, Cappato R, Kim YH, Saad EB, Aguinaga L, *et al.* 2017 HRS/EHRA/ECAS/APHRS/SOLAECE expert consensus statement on catheter and surgical ablation of atrial fibrillation. *Europace* 2018;**20**:e1–160.

22. Knopp H, Halm U, Lamberts R, Knigge I, Zachäus M, Sommer P, *et al.* Incidental and ablation-induced findings during upper gastrointestinal endoscopy in patients after ablation of atrial fibrillation: A retrospective study of 425 patients. *Heart Rhythm*; 2014;**11**:574–8.

23. Cochet H, Nakatani Y, Sridi-Cheniti S, Cheniti G, Ramirez FD, Nakashima T, *et al.* Pulsed field ablation selectively spares the oesophagus during pulmonary vein isolation for atrial fibrillation. *Europace*; 2021;**23**:1391–9.

24. Nakatani Y, Sridi-Cheniti S, Cheniti G, Ramirez FD, Goujeau C, André C, *et al.* Pulsed field ablation prevents chronic atrial fibrotic changes and restrictive mechanics after catheter ablation for atrial fibrillation. *Europace*; 2021;**23**:1767–76.

**Tables**

**Table 1: Baseline (A) and procedure (B) characteristics (n = 40)**

| **A) Baseline Characteristics** | **Value** |
| --- | --- |
| Age (years) | 62 ± 9 |
| Male gender | 28 (70) |
| BMI (kg/m²) | 28 (25-29) |
| Hypertension | 24 (60) |
| Diabetes | 1 (2.5) |
| Stroke or TIA | 3 (7.5) |
| LA diameter (mm) | 41 ± 4 |
| LVEF (%) | 58 ± 4 |
| *Atrial fibrillation* |  |
| - paroxysmal | 25 (62.5) |
| - persistent | 15 (37.5) |
| **B) Procedure Characteristics** | **Value** |
| Procedure time (min) | 110 (100-124) |
| Fluoro time (min) | 16 (14-24) |
| Fluoro dose (cGcm²) | 125 (68-196) |
| Pre map time (min) | 12 (11-16) |
| PFA catheter dwell time (min) | 33 (28-41) |
| Post map time (min) | 13 (10-17) |
| Points pre map | 5469 ± 1822 |
| Points post map | 6809 ± 2769 |
| 35 mm PFA catheter | 9 (22.5) |
| *PV size (mm)* |  |
| - LSPV | 23 (20-25) |
| - LIPV | 22 (20-25) |
| - RSPV | 24 (21-26) |
| - RIPV | 23 (20-25) |
| Common os | 9 (22.5) |

Data is displayed as n (%), mean ± SD or median (IQR); BMI = body mass index, LA = left atrium, LIPV = left inferior pulmonary vein, LSPV = left superior pulmonary vein, LVEF = left ventricular ejection fraction, PFA = pulsed field ablation, PV = pulmonary vein, RIPV = right inferior pulmonary vein, RSPV = right superior pulmonary vein, TIA = transient ischemic attack.

**Table 2: Qualitative analysis: Frequency of insufficient (A) and enlarged (B) isolation areas**

| **A) Frequency of *insufficient* isolation areas per antral PV segments (n = 40)** | | | | | | | | | | | | |  |
| --- | --- | --- | --- | --- | --- | --- | --- | --- | --- | --- | --- | --- | --- |
|  | | | *Left PVs* | | | | | *Right PVs* | | | | |  |
| PV segment | | | n (%) | p | | | | n (%) | p | | | |  |
| 1 | Sup. | | 18 (45.0) | * | | 0.285 | | 15 (37.5) | * | | 1 | |  |
| 2 | Ant. | | 26 (65.0) | 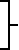 | | **0.019** | | 14 (35.0) | 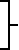 | | 1 | |  |
| 3 | Post. | | 7 (17.5) |  |  |  |  | 15 (37.5) |  |  |  |  |  |
| 4 | Ant. | | 27 (67.5) | 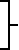 | | **<0.001** | | 8 (20.0) | 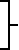 | | 0.375 | |  |
| 5 | Post. | | 1 (2.5) |  |  |  |  | 5 (12.5) |  |  |  |  |  |
| 6 | Ant. | | 25 (62.5) | 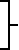 | | **<0.001** | | 12 (30.0) | 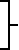 | | 0.065 | |  |
| 7 | Post. | | 2 (5.0) |  |  |  |  | 5 (12.5) |  |  |  |  |  |
| 8 | Ant. | | 31 (77.5) | 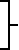 | | **<0.001** | | 16 (40.0) | 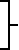 | | 0.302 | |  |
| 9 | Post. | | 3 (7.5) |  |  |  |  | 11 (27.5) |  |  |  |  |  |
| 10 | Inf. | | 13 (32.5) | * | | 0.285 | | 17 (42.5) | * | | 1 | |  |
| **B) Frequency of *enlarged* isolation areas per LA region (n = 38)** | | | | | | | | | | | | | |
|  | | *Left LA* | | | | | | *Right LA* | | | | | |
| LA region | | n (%) | | | p | | | n (%) | | p | | | |
| Roof | | 37 (97.4) | | | 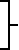 | | **<0.001** | 34 (89.5) | | 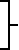 | | 0.289 | |
| Side wall | | 19 (50.0) | | |  |  |  | 29 (76.3) | |  |  |  |  |
| Posterior wall | | 38 (100) | | | 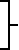 | | **<0.001** | 35 (92.1) | | 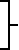 | | **0.004** | |
| Anterior wall | | 20 (52.6) | | |  |  |  | 26 (68.4) | |  |  |  |  |

Data is displayed as n (%); LA = left atrium, PV = pulmonary vein, Sup. = superior, Ant. = anterior, Post. = posterior, Inf. = inferior.

**Table 3: Quantitative analysis: Extent of insufficient (A), enlarged (B), and calculated (C) isolation areas**

| **A) Extent of *insufficient* isolation areas per antral PV segments (n = 40)** | | | | | | | | | | | | | | | | | | | | | |  |
| --- | --- | --- | --- | --- | --- | --- | --- | --- | --- | --- | --- | --- | --- | --- | --- | --- | --- | --- | --- | --- | --- | --- |
|  | | | *Left PVs* | | | | | | | | | *Right PVs* | | | | | | | | | |  |
| Antral PV segment | | Total area (cm²) | | | | Insufficient isolation area (cm²) | | | p | | | | Total area (cm²) | | Insufficient isolation area (cm²) | | | p | | |  |  |
| 1 | Sup. | | 0.6 (0.5-0.7) | | | | 0.1 (0.1-0.6) | | * | | 0.102 | | 0.6 (0.5-0.7) | | 0.2 (0.1-0.5) | | | * | 1 | |  |  |
| 2 | Ant. | | 0.6 (0.5-0.5) | | | | 0.4 (0.3-0.5) | | 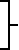 | | **0.028** | | 0.6 (0.5-0.7) | | 0.2 (0.2-0.3) | | | 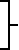 | 0.156 | |  |  |
| 3 | Post. | | 0.5 (0.5-0.6) | | | | 0.1 (0.1-0.4) | |  |  |  |  | 0.6 (0.5-0.6) | | 0.2 (0.1-0.3) | | |  |  |  |  |  |
| 4 | Ant. | | 0.8 (0.6-1.0) | | | | 0.4 (0.3-0.5) | | 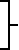 | | **<0.001** | | 0.9 (0.7-1.0) | | 0.2 (0.1-0.4) | | | 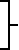 | 0.715 | |  |  |
| 5 | Post. | | 0.7 (0.6-0.9) | | | | 0.1 (0.1-0.1) | |  |  |  |  | 0.9 (0.7-1.0) | | 0.3 (0.2-0.4) | | |  |  |  |  |  |
| 6 | Ant. | | 0.7 (0.6-0.9) | | | | 0.3 (0.1-0.4) | | 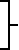 | | **<0.001** | | 0.9 (0.7-0.9) | | 0.3 (0.2-0.6) | | | 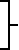 | 0.180 | |  |  |
| 7 | Post. | | 0.7 (0.6-0.9) | | | | 0.1 (0.1-0.1) | |  |  |  |  | 0.8 (0.7-1.0) | | 0.1 (0.1-0.4) | | |  |  |  |  |  |
| 8 | Ant. | | 0.6 (0.5-0.7) | | | | 0.4 (0.1-0.6) | | 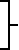 | | **<0.001** | | 0.6 (0.5-0.7) | | 0.4 (0.3-0.5) | | | 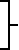 | 0.167 | |  |  |
| 9 | Post. | | 0.5 (0.5-0.6) | | | | 0.1 (0.1-0.1) | |  |  |  |  | 0.6 (0.5-0.7) | | 0.2 (0.1-0.3) | | |  |  |  |  |  |
| 10 | Inf. | | 0.6 (0.5-0.7) | | | | 0.2 (0.1-0.5) | | * | | 0.102 | | 0.7 (0.6-0.7) | | 0.2 (0.1-0.4) | | | * | 1 | |  |  |
| **B) Extent of *enlarged* isolation area per LA region (n = 38)** | | | | | | | | | | | | | | | | | | | | | | |
|  | | | | | *Left LA* | | | | | | | *Right LA* | | | | | | | | | | |
| LA region | | | | Total area (cm²) | | | Enlarged isolation area (cm²) | p | | | | Total area (cm²) | | Enlarged isolation  area (cm²) | | | | p | | | | |
| Roof | | | 5.2 (4.0-6.4) | | | | 2.7 (1.3-3.5) | 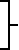 | | **<0.001** | | 4.5 (3.8-6.2) | | 1.4 (0.6-2.0) | | | | 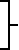 | | 0.467 | | |
| Side wall | | | 3.6 (3.0-4.5) | | | | 0.1 (0.0-0.7) |  |  |  |  | 3.8 (3.5-4.9) | | 1.0 (0.2-2.0) | | | |  |  |  |  |  |
| Posterior wall | | | 7.8 (6.8-9.0) | | | | 2.1 (0.9-3.5) | 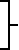 | | **<0.001** | | 7.4 (6.2-8.4) | | 1.1 (0.6-2.0) | | | | 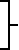 | | **0.002** | | |
| Anterior wall | | | 5.7 (4.5-6.9) | | | | 0.3 (0.0-0.9) |  |  |  |  | 5.9 (4.2-6.6) | | 0.6 (0.0-1.1) | | | |  |  |  |  |  |
| **C) Extent of *calculated* isolation areas (n = 38)** | | | | | | | | | | | | | | | | | | | | | | |
|  | | | | | | | | *Total* | | | | *Left LA* | | | | *Right LA* | *p* | | | | | |
| Circumferential PV isolation area (cm²) | | | | | | | | 22.2 ± 5.6 | | | | 11.2 ± 3.3 | | | | 11.0 ± 3.7 | 0.676 | | | | | |
| Total posterior wall area (cm²) | | | | | | | | 31.2 ± 6.5 | | | | 15.9 ± 3.5 | | | | 15.3 ± 3.2 | **0.015** | | | | | |
| PW PV isolation area (cm²) | | | | | | | | 13.2 ± 4.0 | | | | 7.7 ± 2.9 | | | | 5.5 ± 2.0 | **<0.001** | | | | | |
| Non-ablated PW area (cm²) | | | | | | | | 18.1 ± 5.8 | | | | 8.2 ± 3.2 | | | | 9.8 ± 3.2 | **0.001** | | | | | |

Data is displayed as median (IQR) or mean ± SD; Ant. = anterior, Inf. = inferior, LA = left atrium, Post. = posterior, PV = pulmonary vein, PW = posterior wall, Sup. = superior.

**Figures**

**Figure 1: Frequency of insufficient isolation area per antral PV segments (n = 40)**


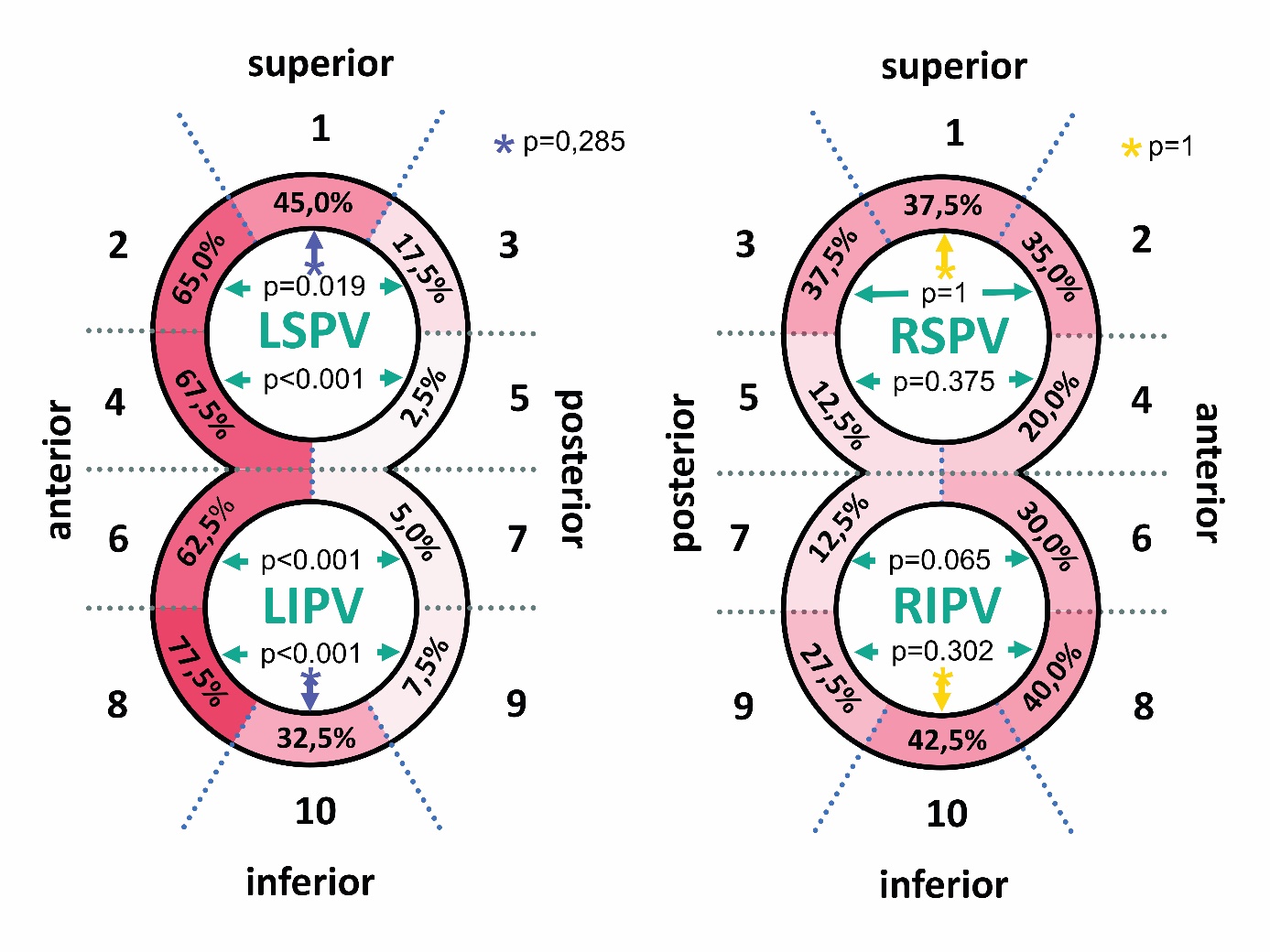


Data is displayed as percentage; LIPV = left inferior pulmonary vein, LSPV = left superior pulmonary vein, PV = pulmonary veins, RIPV = right inferior pulmonary vein, RSPV = right superior pulmonary vein.

**Figure 2: Examples of resulting isolation areas**


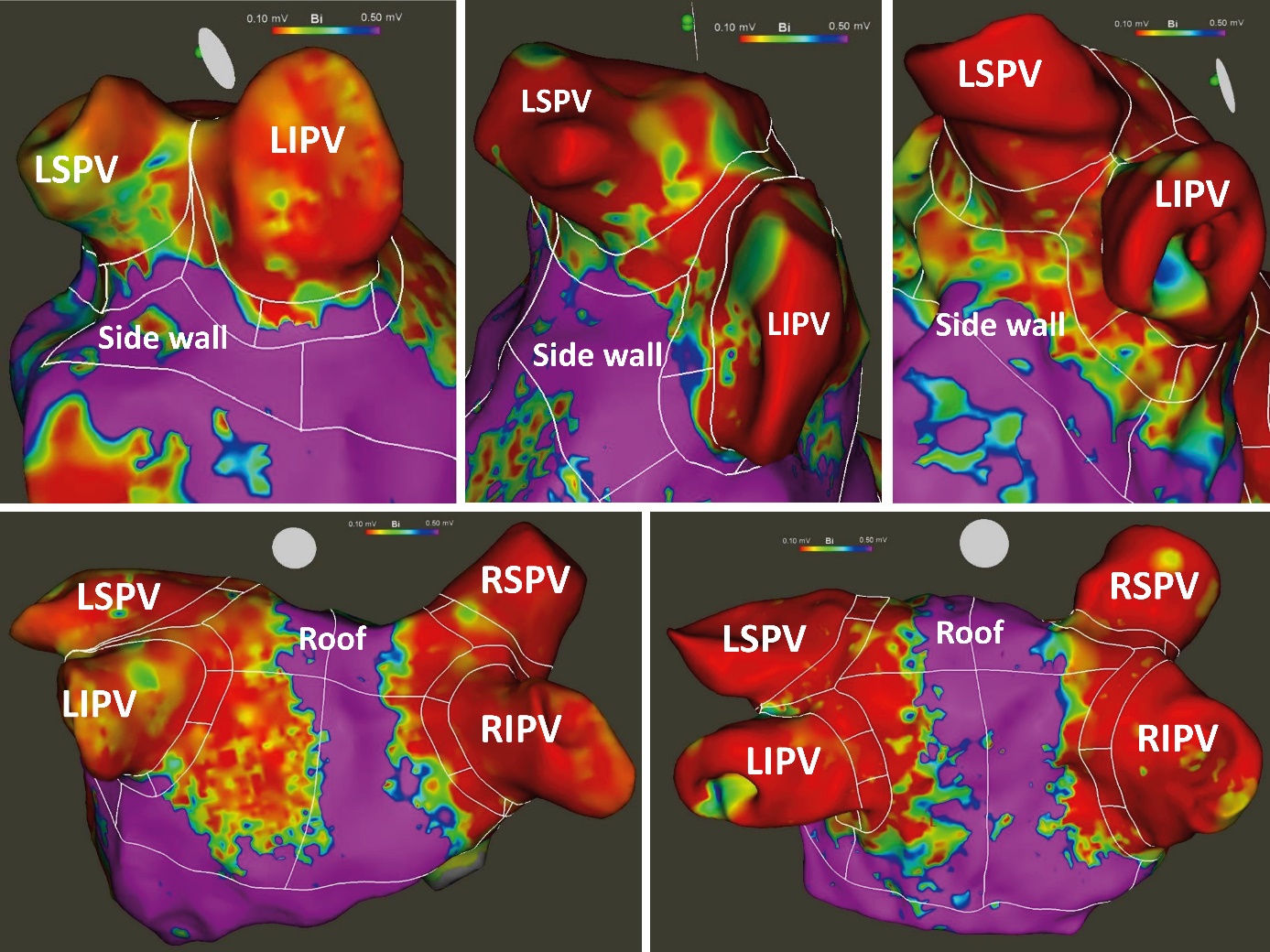


(A+B) Insufficient isolation of the left anterior antral PV segments; (C) Sufficient isolation of the left anterior antral PV segments; (D) Enlarged isolation of the posterior wall; (E) Sufficient circumferential antral PV isolations areas with acceptable degree of enlarged ablation.

LIPV = left inferior pulmonary vein, LSPV = left superior pulmonary vein, PV = pulmonary veins, RIPV = right inferior pulmonary vein, RSPV = right superior pulmonary vein.

**Figure 3: Extent of insufficient isolation area per antral *left* and *right* PV segments (n = 40)**


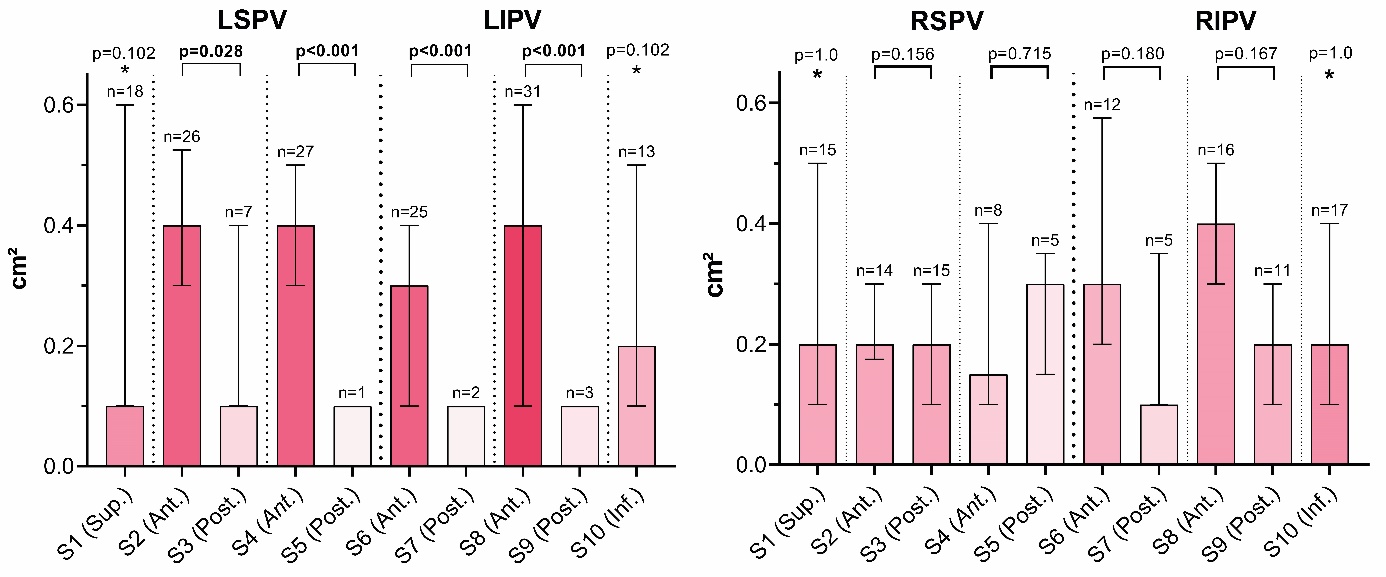


Data is displayed as median (IQR); LIPV = left inferior pulmonary vein, LSPV = left superior pulmonary vein, PV = pulmonary veins, RIPV = right inferior pulmonary vein, RSPV = right superior pulmonary vein, S = segment.

**Figure 4: Extent of enlarged isolation area per LA region (n = 38)**

**
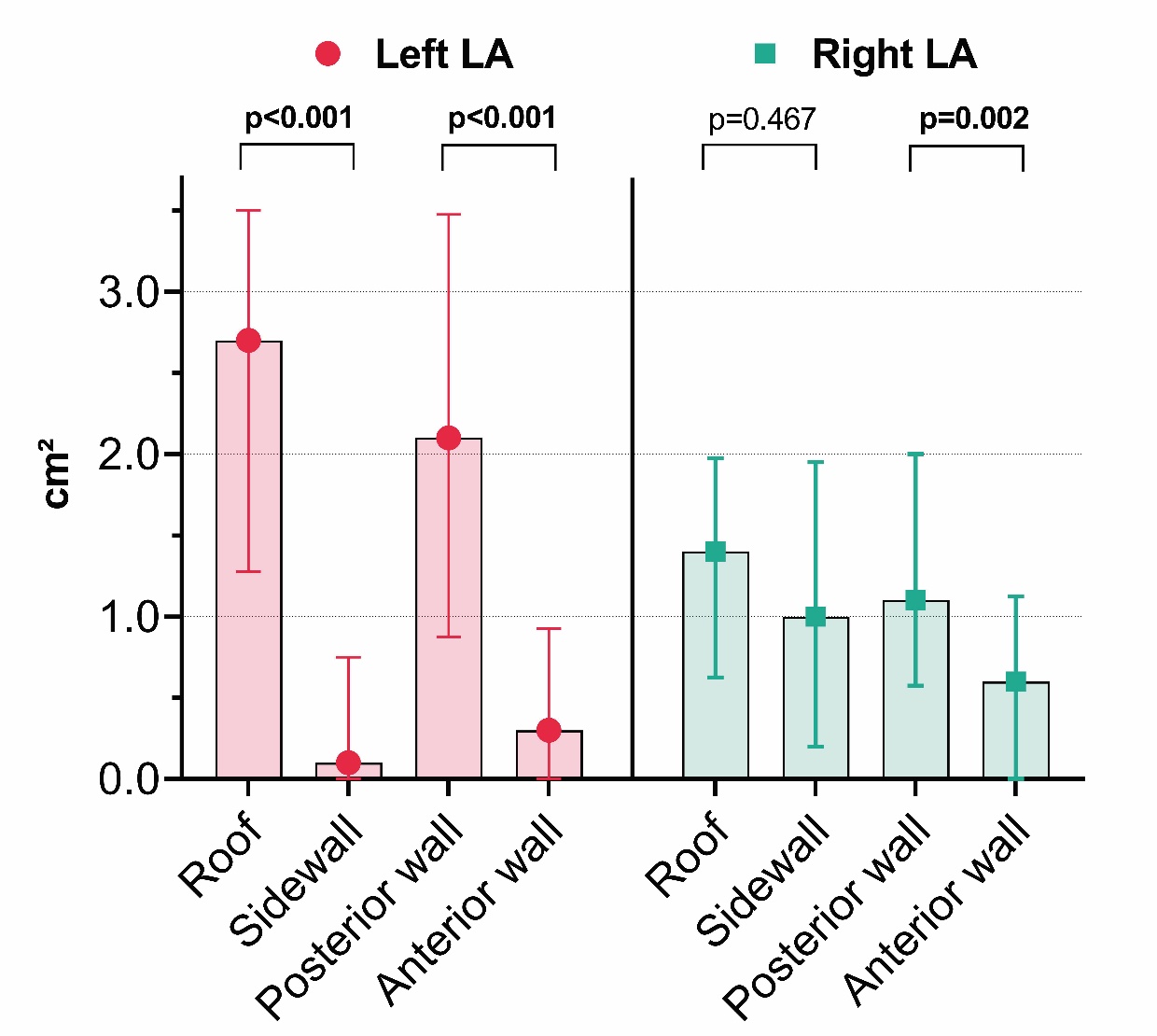
**

Data is displayed as median (IQR); LA = left atrium.
