# Supplementary material for: Characterization of circumferential antral pulmonary vein isolation areas resulting from pulsed-field catheter ablation
Source: Europace. 2022 Jul 19;25(1):65–73. doi: 10.1093/europace/euac111 (PMC10103571; doi:10.1093/europace/euac111)
Supplement: euac111_Supplementary_Data [file euac111_supplementary_data.zip › Supplement Rv2 - PFA-PVI Area.docx]

**Supplement**

**Supplementary Table 1: Mapping system configurations**

| **Mapping system configurations:** |  |
| --- | --- |
| - Position stability | “4” |
| - Local activation time stability | “3” |
| - Cycle length variability | “-100 to +100 ms” of the mean cycle length of the present rhythm |
| - Fill & color threshold | “5” |
| - Respiratory compensation | “activated” |
| - Regional detail level   - When mapping the PVs and the adjacent circumferential antrum | “20” |
| - Regional detail level   - When mapping the remainder of the LA | “18” |

LA = left atrium

**Supplementary Table 2: Calculation of isolation areas**

| **Variable** | **Calculation** |
| --- | --- |
| Circumferential PV isolation area | = isolation area of all antral PV segments [S1-S10] + enlarged isolation area of roof, PW, SW, and AW for left and right PV/LA, respectively. |
| Total PW area | = total area of antral PV segments S3+S5+S7+S9 + total area of roof + PW |
| PW PV isolation area | = isolation area of antral PV segments S3+S5+S7+S9 + total area of roof + PW |
| Non-ablated PW area | = total PW area – PW isolation area |

PV = pulmonary vein, PW = posterior wall, SW = sidewall, AW = anterior wall, LA = left atrium, S = segment

**Supplementary Figure 1: Ten-segment model of left and right PVs**

**
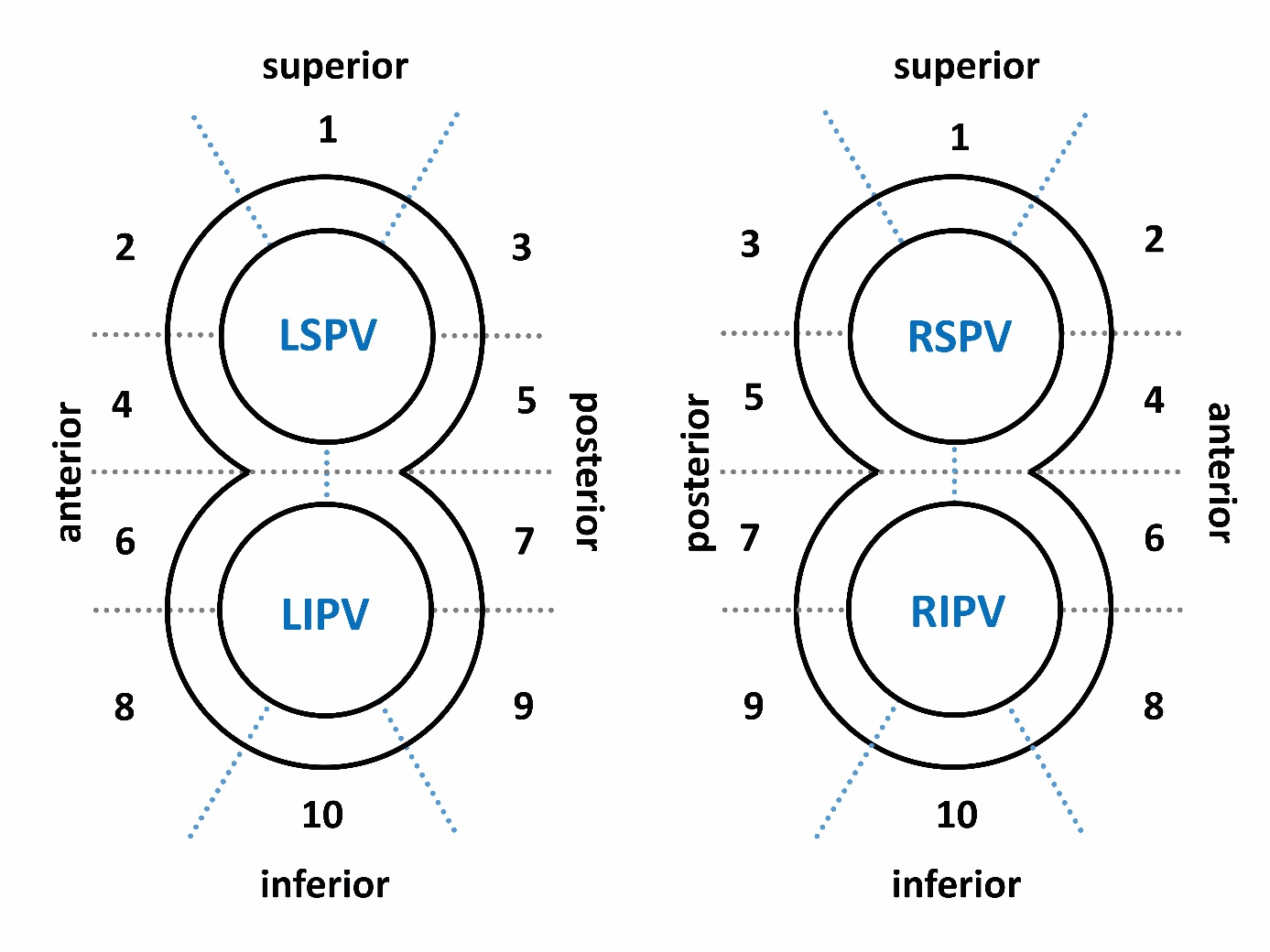
**

PV = pulmonary vein, LSPV = left superior pulmonary vein, LIPV = left inferior pulmonary vein, RSPV = right superior pulmonary vein, RIPV = right inferior pulmonary vein.

**Supplementary Figure 2: Eight-region model of the left atrium (anterior right lateral view)**


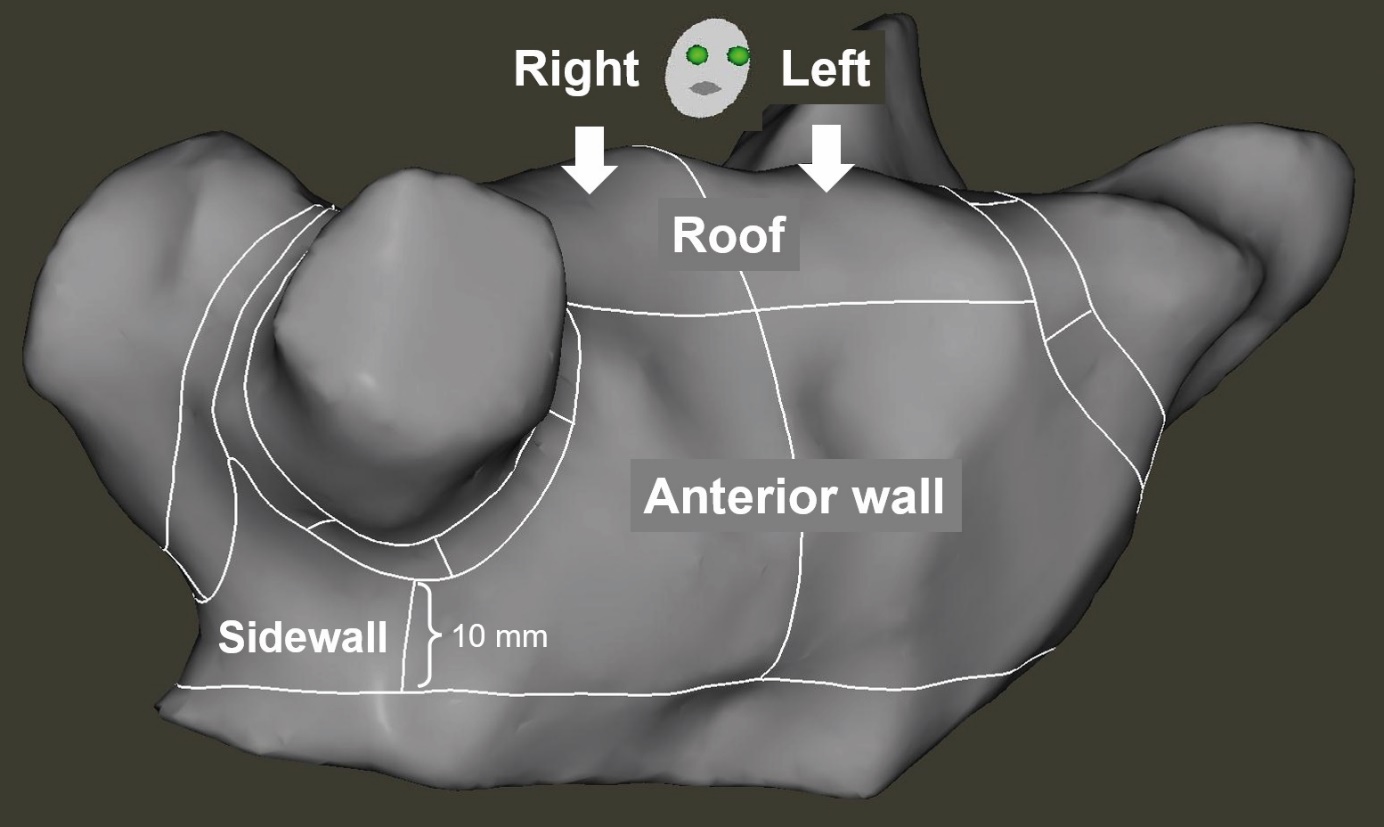


**Supplementary Figure 3: Eight-region model of the left atrium (posterior left lateral view)**


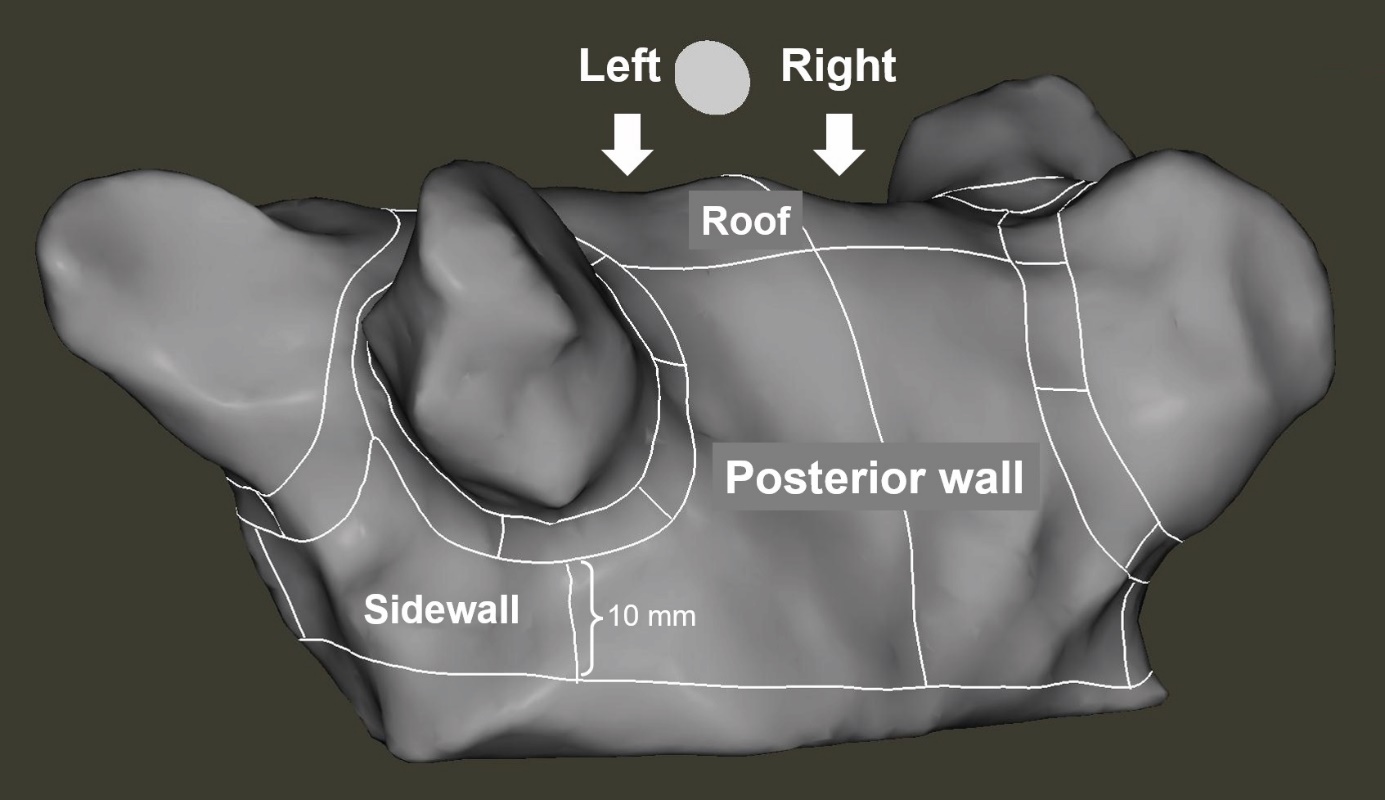


**Authors’ individual contribution to the manuscript**

Marius Bohnen and Heiko Lehrmann planned and designed the study. Marius Bohnen and Heiko Lehrmann acquired the data, interpreted the data and wrote the manuscript. Marius Bohnen analyzed the data. Reinhold Weber, Jan Minners, Martin Eichenlaub, Amir Jadidi, Franz-Josef Neumann and Thomas Arentz interpreted the data, revised the manuscript and substantially contributed to the inaugural draft. All authors approved the final submitted version.

**Study Funding**

The funder (University Hospital Freiburg) had no role in the design of the study; in the collection, analyses, or interpretation of data; in the writing of the manuscript, or in the decision to publish the results. This study was performed and designed without the input or support of any pharmaceutical or medical device company, or other commercial interest.

**Appendix 1: Authors**

| **Name** | **Location** | **Role** | **Contribution** |
| --- | --- | --- | --- |
| Marius Bohnen, MD | University Hospital Freiburg, Germany | Author, data management and statistician | Major role in the acquisition of data; designed and conceptualized study; analyzed the data; interpreted the data; drafted the manuscript |
| Reinhold Weber, MD | University Hospital Freiburg, Germany | Author | Interpreted the data; revised the manuscript for intellectual content |
| Jan Minners, MD, PhD | University Hospital Freiburg, Germany | Author | Interpreted the data; revised the manuscript for intellectual content |
| Amir Jadidi, MD | University Hospital Freiburg, Germany | Author | Interpreted the data; revised the manuscript for intellectual content |
| Martin Eichenlaub, MD | University Hospital Freiburg, Germany | Author | Interpreted the data; revised the manuscript for intellectual content |
| Franz-Josef Neumann, MD | University Hospital Freiburg, Germany | Author | Interpreted the data; revised the manuscript for intellectual content |
| Thomas Arentz, MD | University Hospital Freiburg, Germany | Author | Interpreted the data; revised the manuscript for intellectual content |
| Heiko Lehrmann, MD | University Hospital Freiburg, Germany | Author and data management | Major role in the acquisition of data; designed and conceptualized study; interpreted the data; drafted the manuscript |
