# Supplementary figures and images for: Characterization of circumferential antral pulmonary vein isolation areas resulting from pulsed-field catheter ablation
Source: Europace. 2022 Jul 19;25(1):65–73. doi: 10.1093/europace/euac111 (PMC10103571; doi:10.1093/europace/euac111)

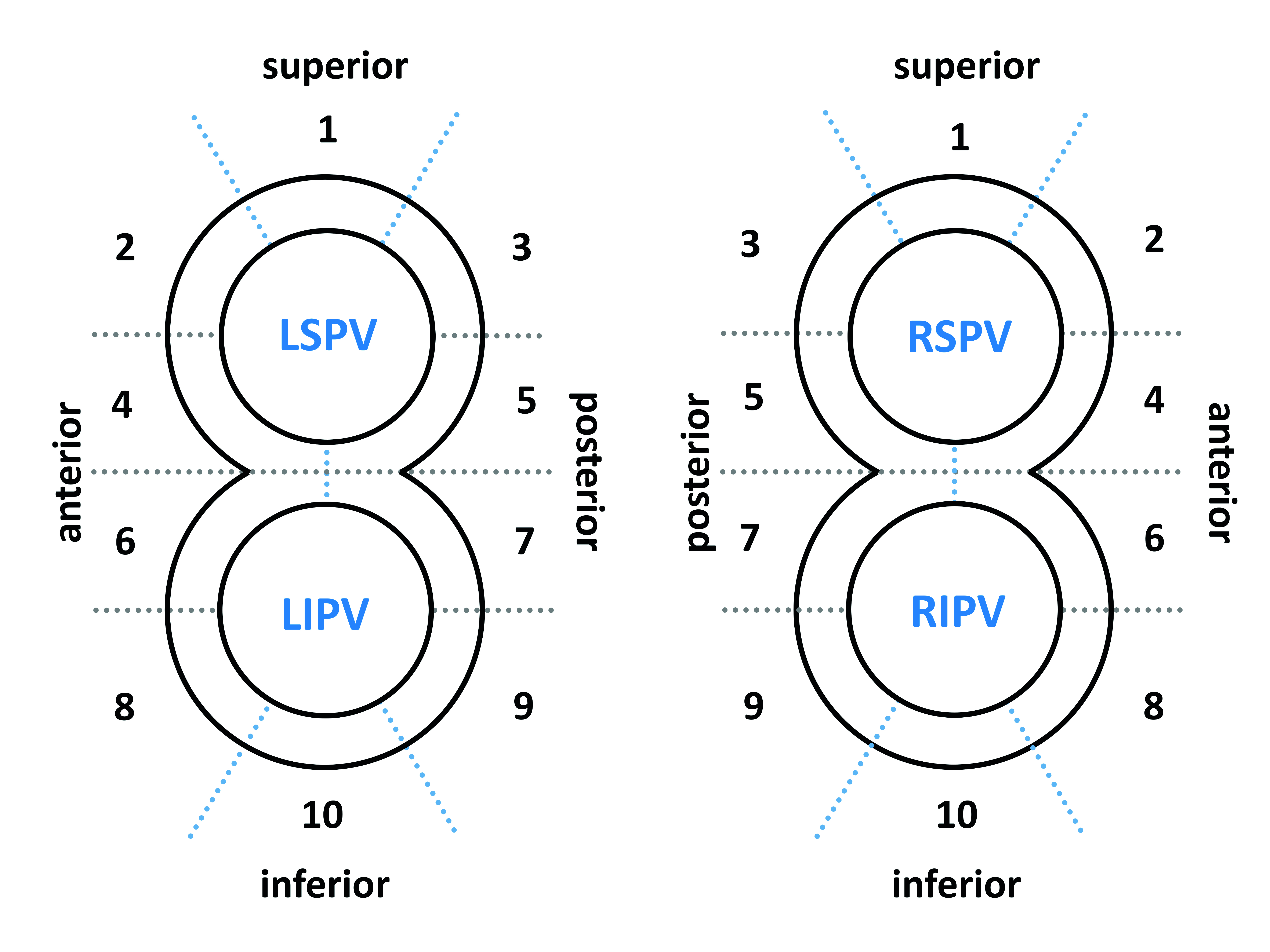

Supplement: euac111_Supplementary_Data [file euac111_supplementary_data.zip › Suppl Figure 1.jpg]

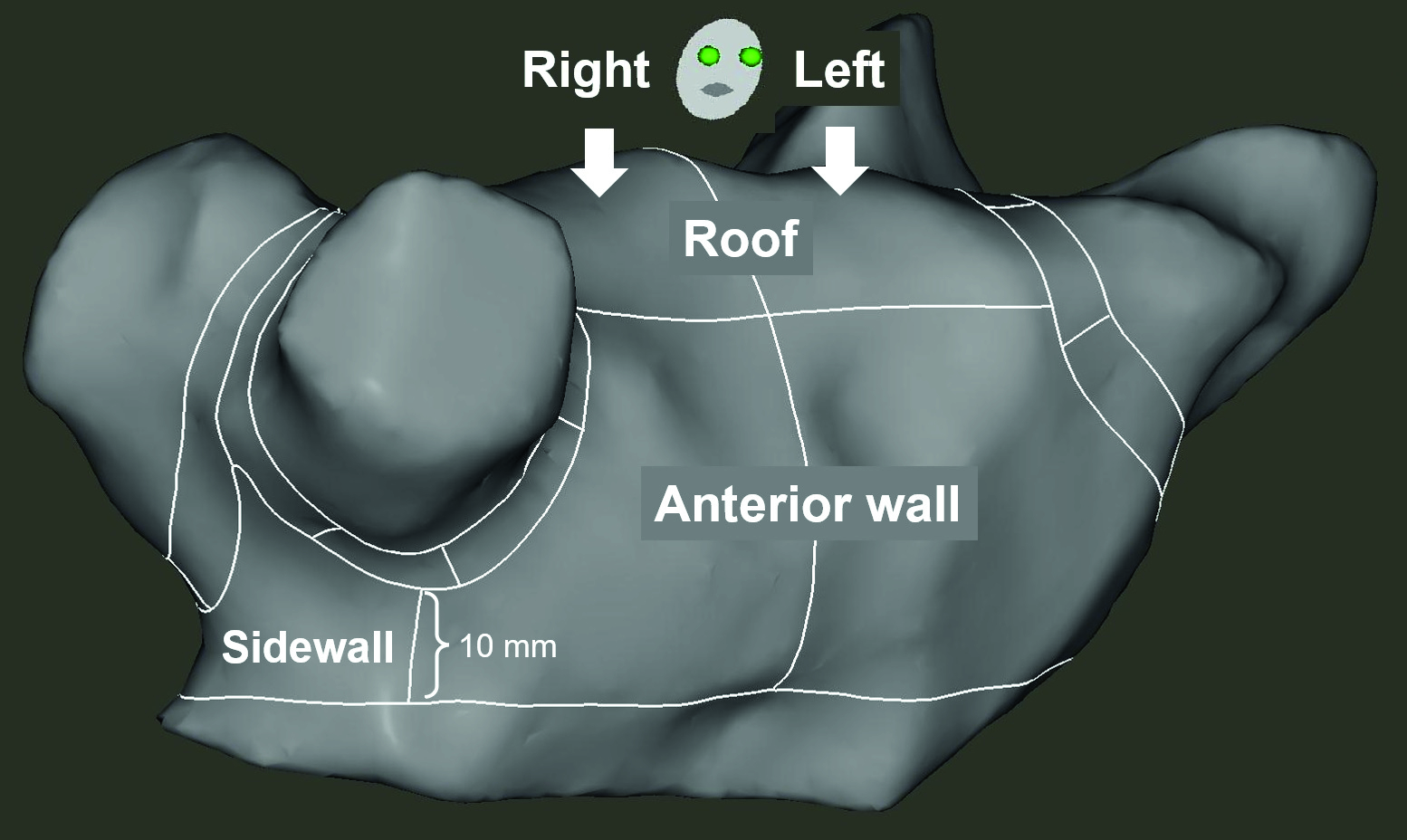

Supplement: euac111_Supplementary_Data [file euac111_supplementary_data.zip › Suppl Figure 2.jpg]

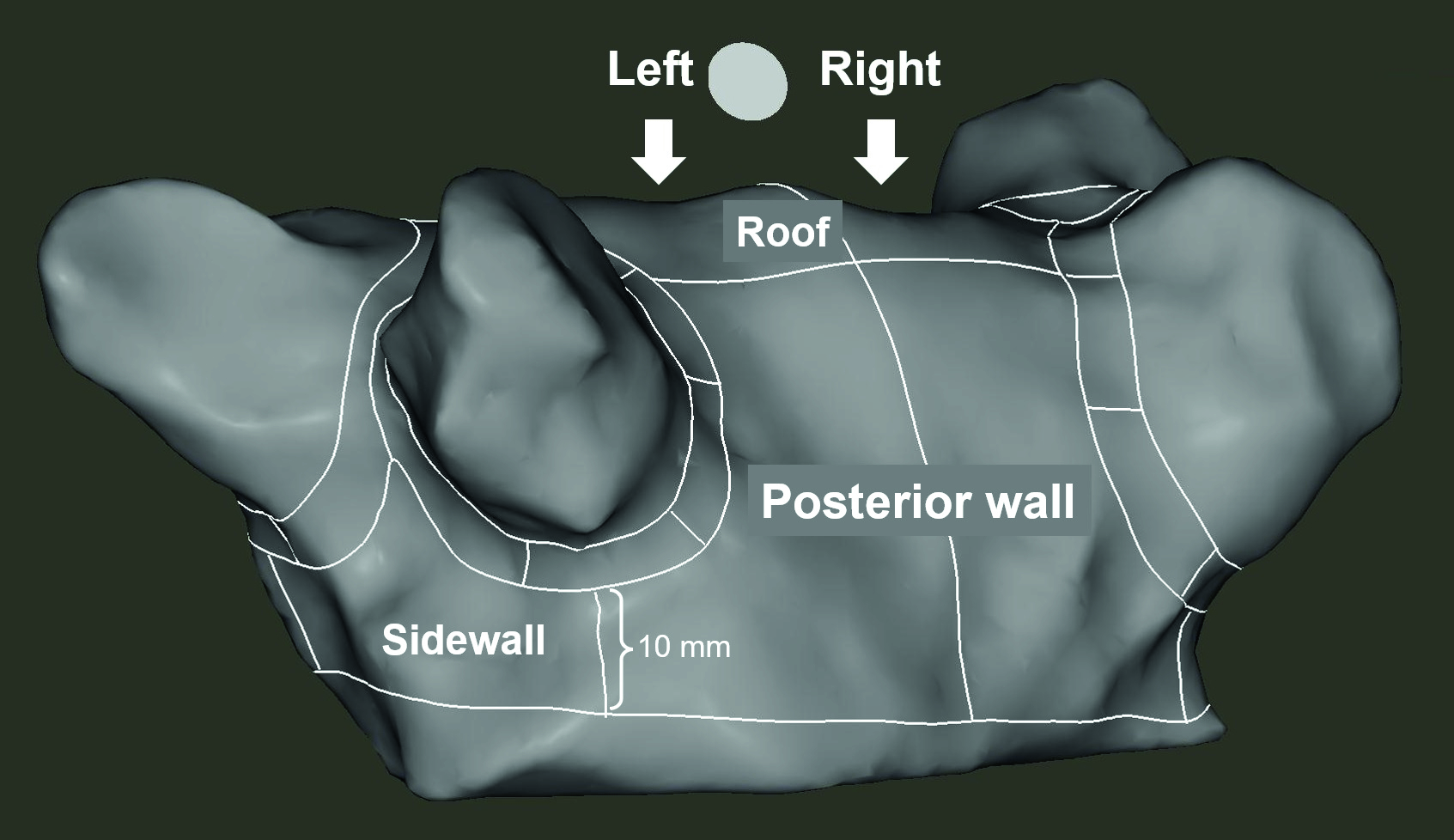

Supplement: euac111_Supplementary_Data [file euac111_supplementary_data.zip › Suppl Figure 3.jpg]
